# Root Adaptation via Common Genetic Factors Conditioning Tolerance to Multiple Stresses for Crops Cultivated on Acidic Tropical Soils

**DOI:** 10.3389/fpls.2020.565339

**Published:** 2020-11-12

**Authors:** Vanessa A. Barros, Rahul Chandnani, Sylvia M. de Sousa, Laiane S. Maciel, Mutsutomo Tokizawa, Claudia T. Guimaraes, Jurandir V. Magalhaes, Leon V. Kochian

**Affiliations:** ^1^Embrapa Maize and Sorghum, Sete Lagoas, Brazil; ^2^Departamento de Biologia Geral, Universidade Federal de Minas Gerais, Belo Horizonte, Brazil; ^3^Global Institute for Food Security, University of Saskatchewan, Saskatoon, SK, Canada

**Keywords:** acid soils, aluminum toxicity, aluminum tolerance, phosphorus deficiency, phosphorus efficiency, drought resistance, transcription factor, signaling

## Abstract

Crop tolerance to multiple abiotic stresses has long been pursued as a Holy Grail in plant breeding efforts that target crop adaptation to tropical soils. On tropical, acidic soils, aluminum (Al) toxicity, low phosphorus (P) availability and drought stress are the major limitations to yield stability. Molecular breeding based on a small suite of pleiotropic genes, particularly those with moderate to major phenotypic effects, could help circumvent the need for complex breeding designs and large population sizes aimed at selecting transgressive progeny accumulating favorable alleles controlling polygenic traits. The underlying question is twofold: do common tolerance mechanisms to Al toxicity, P deficiency and drought exist? And if they do, will they be useful in a plant breeding program that targets stress-prone environments. The selective environments in tropical regions are such that multiple, co-existing regulatory networks may drive the fixation of either distinctly different or a smaller number of pleiotropic abiotic stress tolerance genes. Recent studies suggest that genes contributing to crop adaptation to acidic soils, such as the major Arabidopsis Al tolerance protein, AtALMT1, which encodes an aluminum-activated root malate transporter, may influence both Al tolerance and P acquisition via changes in root system morphology and architecture. However, *trans*-acting elements such as transcription factors (TFs) may be the best option for pleiotropic control of multiple abiotic stress genes, due to their small and often multiple binding sequences in the genome. One such example is the C2H2-type zinc finger, AtSTOP1, which is a transcriptional regulator of a number of Arabidopsis Al tolerance genes, including *AtMATE* and *AtALMT1*, and has been shown to activate *AtALMT1*, not only in response to Al but also low soil P. The large WRKY family of transcription factors are also known to affect a broad spectrum of phenotypes, some of which are related to acidic soil abiotic stress responses. Hence, we focus here on signaling proteins such as TFs and protein kinases to identify, from the literature, evidence for unifying regulatory networks controlling Al tolerance, P efficiency and, also possibly drought tolerance. Particular emphasis will be given to modification of root system morphology and architecture, which could be an important physiological “hub” leading to crop adaptation to multiple soil-based abiotic stress factors.

## Introduction

Acidic soils (soils pH < 5.5) are quite extensive worldwide, comprising up to 50% of the world’s potentially arable lands (Von Uexküll and Mutert, 1995). As the acidic weathered soils are particularly prominent in the humid tropics and subtropics where many developing countries in sub-Saharan Africa and Asia are located, and food production must keep pace with population growth (Godfray et al., 2010), acidic soils are a major constraint for developing world agriculture The two most significant limitations to crop production on acid soils from the plant nutrition perspective are aluminum (Al) toxicity and phosphorus (P) deficiency (Kochian et al., 2015). Both arise from the unique chemical properties of highly weathered acid soils. Aluminum is the most abundant metal in the earth’s crust as it is a major component of clays, as aluminosilicates. At soil pH values of pH 5.5 and below, Al^3+^ ions are solubilized into the soil solution. Al^3+^ is quite toxic to roots, inhibiting both root elongation and root meristem cell division (see, for example, Kochian, 1995; Ma et al., 2001, and references therein). This results in major reductions in yields due to insufficient water and mineral nutrient uptake by the root systems. Low-P soil levels and availability also arise from the chemical properties of acidic soils as soil weathering exposes Al and Fe oxides/hydroxides on the surface of clay minerals, which bind soil P (as the phosphate anion) tightly, reducing its bioavailability (Marschner, 1995). Soils with low P availability will be designated henceforth as low-P soils for brevity. The third related stress we address in this review is drought stress, which is found on all soil types, including acidic soils. The unique aspect to acidic soils is that crop adaptation to drought on those soils requires that the plants be both Al tolerant to maintain a healthy root system to facilitate water absorption, along with specific adaptations to drought which are ubiquitously found in crop species on all soil types. Because crops acidic soils have had to adapt to all three stresses simultaneously, it is not surprising that especially in recent literature common features in adaptation to these three abiotic stresses are being uncovered. This is the theme we address in this review.

In searching for classes of genes involved in mediating resistance concurrently to these three stresses, it is more likely that “upstream” genes that control regulatory and signaling networks such as transcription factors (TFs), kinases and phosphatases would be more likely candidates than structural genes such as root plasma membrane transporters that mediate efflux of Al-binding organic acid anions that have been shown to be involved in crop Al tolerance (Ma et al., 2001; Ryan et al., 2001; Kochian et al., 2004, 2015). Regulatory genes, such as transcription factors, bind to very small *cis* elements in the promoter region. Depending also on more complex aspects such as chromatin structure, this gives TFs potential for promiscuous binding to many targets, giving rise to complex regulatory circuits. A good example of how the promiscuity of TF binding sites can impact evolutionary adaptation is presented in Pougach et al. (2014), where they show that duplication of a transcription factor gene allowed the emergence of two independent regulatory circuits in yeast. Since TFs are often regulators of response to multiple stresses, they are excellent candidates for breeding programs searching for pleiotropic control of co-existing stresses in acidic soils such as Al toxicity, low P availability, and drought (Baillo et al., 2019).

In this review, we have focused on signaling/regulatory proteins such as TFs and protein kinases to identify, from the literature, evidence for unifying regulatory networks controlling Al tolerance, P efficiency and also possibly drought tolerance. Particular emphasis will be given to modification of root system morphology and architecture, which could be an important physiological “hub” leading to crop adaptation to multiple soil-based abiotic stress factors.

## Aluminum Toxicity and Tolerance

### Transcriptional Regulation Involved in Al Tolerance

Aluminum (Al) on acidic soils intoxicates root regions involved in root growth (meristem and elongation zone). Cells in these regions are subject to rapid alterations in Al-induced transcription, resulting in the induction of expression of several Al tolerance genes associated with root tip Al exclusion and detoxification mechanisms (Kochian et al., 2015).

Several TFs (TFs) have been reported to be involved in crop Al tolerance, and the majority of these TFs belong to zinc finger and WRKY transcription factor families. Among them, *STOP1* in Arabidopsis and *ART1* in rice are the best characterized TFs regulating Al tolerance. STOP1, a C2H2-type zinc finger transcription factor, was identified via positional cloning of a low-pH-sensitive Arabidopsis mutant. Although *AtSTOP1* expression is not induced by Al, the *stop1*-mutant is also Al hypersensitive (Iuchi et al., 2007). AtSTOP1 has four functional zinc finger domains that bind to a 15-bp long sequence in the *AtALMT1* promoter. *AtALMT1* is the major Arabidopsis Al tolerance gene (Hoekenga et al., 2006), closely related to the primary wheat Al tolerance gene, *TaALMT1* (Sasaki et al., 2004). These two ALMT genes and similar ones in other plant species encode root cell plasma membrane Al-activated malate efflux transporters that are one of the key genes involved in root Al exclusion via release of Al-binding organic acid anions. Mutations in the STOP1 binding sites and in AtSTOP1 zinc finger domains critically suppress *AtALMT1* expression, indicating that STOP1 binding is essential for *AtALMT1* expression and Al tolerance in Arabidopsis (Tokizawa et al., 2015). Furthermore, AtSTOP1 also regulates the expression of other transporters required for Al tolerance in Arabidopsis, including *AtMATE* (Al-activated citrate transporter) and *AtALS3* (ABC transporter-like protein) (Liu et al., 2009; Sawaki et al., 2009).

*AtSTOP1* is ubiquitously expressed in the root with higher expression in the root tip, and its expression is not affected by Al stress. In turn, AtSTOP1 downstream genes (*AtALMT1*, *AtMATE*, and *AtALS3*) are induced by Al (Liu et al., 2009; Sawaki et al., 2009). These findings suggest that Al might induce *AtSTOP1* regulation at the post-transcriptional/-translational level. Recently, an F-box protein that regulates the level of AtSTOP1 protein, RAE1, was identified in Arabidopsis (Zhang Y. et al., 2019). The authors showed that RAE1 interacts with SKP1, a protein involved in ubiquitination and subsequent proteasomal degradation of target proteins. These two proteins interact to form a functional SCF-type E3 ligase complex, physically binding to STOP1 and driving its degradation via the ubiquitin 26S proteasome. As such, *AtALMT1* and other STOP1 regulated genes, including *AtMATE* and *AtALS3*, are overexpressed in the *rae1* mutant. Interestingly, AtSTOP1 binds to the *RAE1* promoter and positively regulates its expression, indicating that there is a negative feedback loop between *AtSTOP1* and *RAE1*. Finally, the authors suggest that the feedback loop might be important in controlling AtSTOP1 homeostasis, enabling the degradation of accumulated AtSTOP1 after Al stress.

The transcriptional regulation of *AtALMT1* expression by STOP1 is fairly well characterized. However, as stated in Tokizawa et al. (2015), the structure of the *AtALMT1* promoter indicates that other factors may be acting on its expression. The authors identified several *cis*-elements in the *ALMT1* promoter related to: (1) Al-induced early and late expression; (2) root tip-specific expression; and (3) repression of *ALMT1* expression. In addition, it was reported that the transcription factor, *CAMTA2* (Calmodulin binding *trans*-activator 2), binds to the *AtALMT1* promoter at a *cis*-element in a different binding region than STOP1, and appears to be involved in induction of *AtALMT1* expression only in late Al stress. Previously, it was also demonstrated that AtWRKY46 binds to W-box sequences in the *AtALMT1* promoter, repressing its expression in the absence of Al (Ding et al., 2013), indicating that regulation of *AtALMT1* is not restricted to STOP1.

Sharing 41.2% sequence identity with AtSTOP1, the rice homolog, OsART1, was identified by map-based cloning of an Al-sensitive rice mutant. ART1 is also a C2H2-type zinc finger transcription factor involved in the regulation of a number of rice Al responsive genes, but, unlike *AtSTOP1*, it is not responsive to low pH. Microarray analyses showed that OsART1 regulates at least 31 downstream genes in response to Al (Yamaji et al., 2009). This transcription factor directly binds to a GGNVS core sequence in the *OsSTAR1* promoter, which is present in 29 of the 31 ART1-regulated genes (Tsutsui et al., 2011). Some of the ART1-regulated genes have been functionally characterized as being involved in Al tolerance, including a number of transporters mediating Al uptake into the root and the vacuole, a Mg uptake transporter, and an ABC transporter that helps mediate the release of UDP-glucose into the cell wall to possibly minimize Al binding (Huang et al., 2009, 2012; Xia et al., 2010, 2013; Yokosho et al., 2011; Chen et al., 2012). Interestingly, the GGNVS core promoter sequence is also found in the promoter of genes regulated by AtSTOP1, suggesting that STOP1 and ART1 recognize similar DNA sequences (Tokizawa et al., 2015).

STOP1/ART1-like proteins, and their function in the regulation of Al tolerance genes, are broadly conserved among land plant species, including dicots, monocots, woody plants, and even bryophytes (Chen et al., 2013; Sawaki et al., 2014; Fan et al., 2015; Daspute et al., 2018; Huang et al., 2018; Wu et al., 2018; Ito et al., 2019; Kundu et al., 2019). The genome of the moss, *Physcomitrella patens* has a functional STOP1-like protein, and knock out of *PpSTOP1* results in an Al sensitive phenotype, suggesting that plants acquired *STOP1* at a very early time during land adaptation of plants, protecting roots from toxic environments including Al and low pH (Ohyama et al., 2013).

In addition to OsART1, OsWRKY22 also regulates the Al-induced expression of *OsFRDL4*, which encodes the rice root plasma membrane citrate efflux transporter. *OsWRKY22* is rapidly induced by Al and works as a transcriptional activator of the *OsFRDL4* expression via binding to W-box *cis*-elements in the *FRDL4* promoter. OsWRKY22 has not been shown to regulate other *ART1*-regulated genes, however, OsWRKY22 and OsART1 are essential for the full activation of Al-induced *FRDL4* expression and root citrate secretion in rice (Li et al., 2018).

Recently, through QTL mapping, GWAS, and functional analyses, two novel TFs in sorghum were identified, SbWRKY1 and SbZNF1, which positively regulate *SbMATE* expression (Melo et al., 2019). Previously, it was reported that miniature inverted-repeat transposable elements (MITE) in the *SbMATE* promoter play a critical role in its expression, and the number of MITE repeats is strongly correlated with *SbMATE* expression level and Al tolerance in sorghum (Magalhaes et al., 2007), which is consistent with the findings of (Salvi et al., 2007) showing that allelic polymorphisms due to MITE insertions can affect the transcription of regulated genes. These two TFs directly bind to sequences flanking the transposable element and, according to the proposed model, the expanded number of MITE repeats found in Al-tolerant genotypes provides an increased number of binding sites for SbWRKY1 and SbZNF1, resulting in higher sorghum *SbMATE* expression and Al tolerance (Melo et al., 2019). Similar to *SbMATE*, other studies have shown that the diversity of the promoter structures contributes to differences in Al tolerance between tolerant and sensitive genotypes in several crops. Al responsive genes of tolerant accessions of wheat (*TaALMT1*), *Holcus lanatus* (*HlALMT1*) and rice (*OsFRDL4*) carry more STOP1/ART1 binding sites in their promoters and exhibit higher expression levels than the same genes in the respective sensitive accessions (Chen et al., 2013; Tokizawa et al., 2015; Yokosho et al., 2016). These findings indicate that the enrichment of transcription factor binding sites in Al-tolerance gene promoters leads to enhanced transcription factor recruitment, which might explain, at least partially, Al tolerance in several crop species.

In addition to zinc-finger and WRKY TFs, *ASR1* and *ASR5* (Abscisic acid, Stress and Ripening protein 1 and 5) are involved in Al tolerance in rice (Arenhart et al., 2013, 2014, 2016). *ASR5* is induced by Al and binds to the *OsSTAR1* promoter and functions together with OsART1 as transcriptional activators of the *OsSTAR1* expression. This study also demonstrated that *ASR5*-silenced plants impair the expression of two other rice Al tolerance genes, *OsNrat1* and *OsFRDL4*, suggesting that ASR5 is also involved in their transcriptional regulation (Arenhart et al., 2014). Subsequently, it was reported that *ASR5*-silenced plants exhibited a similar Al tolerance phenotype as wild type plants. This was attributed to the transcription factor *ASR1*, which, under the silencing of *ASR5*, is highly induced and regulates ASR5-target genes, including *STAR1*, in a non-preferential manner.

Recently, studies searching for novel regulators of Al resistance have identified TFs related to the modification of the cell wall properties (Li C.X. et al., 2019; Lou et al., 2020). In Arabidopsis, it was found that the *wrky47* mutant has reduced Al tolerance and altered subcellular Al distribution, i.e., increased Al accumulation in symplast, and decreased Al content in the root apoplast. According to the authors (Li C.X. et al., 2019), these effects occur due to the reduction of cell wall Al-binding capacity, conferred by decreased hemicellulose-I cell wall content in the mutant. It was demonstrated that AtWRKY47 directly binds to and activates the expression of genes encoding *EXTENSIN-LIKE PROTEIN (ELP)* and *XYLOGLUCAN ENDOTRANSGLUCOSYLASE-HYDROLASES17 (XTH17)*, that are involved in cell wall modification. Within those, XTH17 works in modifying hemicellulosic polymers during cell expansion (Zhu et al., 2014), and ELP is involved in cell wall extension (Li C.X. et al., 2019). These findings indicate that *WRKY47* is involved in Al resistance by increasing cell wall bind of the rhizotoxic Al^3+^ ion, minimizing its effect on the cell wall and also reducing uptake into the root cytoplasm (Li C.X. et al., 2019). Another study showed that *VuNAR1* (*Vigna umbellata NAC-type Al Responsive1*), a rice bean *NAC* transcription factor, is up-regulated by Al in the root apex (Lou et al., 2020). In this paper, it was demonstrated that VuNAR1 binds to the *AtWAK1* (Arabidopsis wall-associated receptor kinase 1) and *VuWAKL1* (*Vigna umbellata WAK1-like*) promoters, positively regulating their expression (Lou et al., 2020). In Arabidopsis, *WAK1* is rapidly induced by Al, and the *AtWAK1* overexpression increases Al tolerance (Sivaguru et al., 2003). Lou et al. (2020) showed that the phenotype of the *Atwak1* mutant is higher root cell wall pectin content under Al stress, and it is believed that the binding of Al ions to the negatively charged carboxylic acid residues in pectin is involved in one of the components of Al rhizotoxicity, with methylation of the pectin carboxyl groups correlating with reduced Al toxicity (Yang et al., 2008).

### Other Signaling Molecules

We still don’t know how plants sense Al ions to trigger Al-dependent gene regulation. However, several signaling molecules have been identified that appear to be involved in initiating Al-induced transcriptional regulation. For example, Al-induced changes in cytosolic Ca^2+^ and pH (H^+^), have been implicated as sensing/signaling molecules in Al signaling [see review by Kochian et al. (2015) and references therein]. In addition to these ions, several other endogenous species, reactive oxygen species (ROS), phytohormones, and the phosphatidylinositol pathway, appear to be involved in Al signal transduction.

#### Reactive Oxygen Species

Reactive oxygen species including peroxides, superoxide, hydroxyl radical, singlet oxygen are produced in response to a range of stress responses (Banti et al., 2010; Miller et al., 2010; Shahid et al., 2014; Hieno et al., 2019). Biomolecules including lipids, proteins, and DNA/RNA are oxidized by ROS, and this oxidative damage leads to organelle disfunction and programmed cell death (PCD) (Van Breusegem and Dat, 2006; Mittler, 2017). To protect the oxidative stress, plants activate antioxidant systems (i.e., ROS scavenging pathways) and also induce/activate a series of heat shock proteins (HSPs, e.g., molecular chaperon) (Sharma et al., 2012; Driedonks et al., 2015; Mishra et al., 2018; Waszczak et al., 2018). Al toxicity has been shown to trigger ROS, including hydrogen peroxides (H_2_O_2_), and Al/H_2_O_2_-mediated PCD was reported in various plant species (Yamamoto et al., 2002; Sivaguru et al., 2013; Huang et al., 2014). To protect against this, Al induces multiple genes associated with antioxidant production, such as peroxidase and superoxide dismutase, and they play a likely secondary role in Al tolerance (Ezaki et al., 2000; Basu et al., 2001). On the other hand, ROS also can act as signal molecules with the best characterized of these involved in plant defenses against pathogens and pests (see review by Bhattacharjee, 2012, and references therein). With regards to plant Al toxicity and tolerance, Sivaguru et al. (2013) showed there is a strong correlation between Al-induced ROS production and *SbMATE* expression, both temporally and spatially in the sorghum root tip. Subsequently, Kobayashi et al. (2013b) showed that *AtALMT1* and *AtMATE* expression are induced by H_2_O_2_ without Al. However, H_2_O_2_ cannot activate malate release from the roots, suggesting that protein activation of ALMT1 is regulated by a H_2_O_2_-independent pathway. In addition, several proteome analyses of Al stress revealed that several heat shock proteins (HSPs) are induced by Al stress (Zhen et al., 2007; Jiang et al., 2015), and the ER resident chaperon, *AtBIP3*, was identified as a possible Al-tolerance gene which is highly expressed in Al tolerant Arabidopsis accessions (Kusunoki et al., 2017). Interestingly, Enomoto et al. (2019) recently reported that AtSTOP1 directly regulates *AtHSF2A*, which is a master regulator of a series of HSPs, under hypoxic conditions. It is known that hypoxia stress involves ROS-mediated oxidative stress (Blokhina et al., 2003; Schmidt et al., 2018). These results suggest that activation of chaperon proteins including HSPs might be involved in signaling leading to tolerance of Al-induced oxidative stress in plants.

Nitric oxide (NO) is also induced by Al and has been suggested to be involved as a signaling molecule in Al signal transduction. There are several reports describing that Al-induced root growth inhibition is alleviated by application of the NO donor, sodium nitroprusside (Wang and Yang, 2005; Zhang et al., 2011; He et al., 2012). More detailed research into the mechanistic basis for NO-regulated Al stress alleviation is still needed, but it may involve Al tolerance based on the following findings: (1) NO enhancement of antioxidant systems to prevent Al-induced oxidative stress (Wang and Yang, 2005; He et al., 2019), (2) NO modulation of OA metabolism and secretion under Al stress (Yang et al., 2012a,b), and (3) Al induction of endogenous ABA that may be a positive regulator of Al resistance (see phytohormone section below) (He et al., 2012).

#### Phytohormones

The root apex is the primary site of Al toxicity, and one of most active sites in the plant for phytohormone signaling (Ryan et al., 1993; Jung and McCouch, 2013). Auxin (i.e., Indole-3-acetic acid [IAA]) is a key regulator for plant root growth and development (Overvoorde et al., 2010). An appropriate auxin gradient with a maximal auxin gradient in the root apex are essential for continuous root growth (Petersson et al., 2009). Several membrane-localized PIN-FORMED (PIN) proteins, which are auxin-efflux transport proteins, play a major role in the regulation of the formation and maintenance of this gradient (Wiśniewska et al., 2006; Grieneisen et al., 2007). Al toxicity disturbs this auxin gradient in the root apex (Kollmeier et al., 2000; Shen et al., 2008); moreover, Al interferes with the appropriate membrane localization of PIN2 in Arabidopsis root tip cells (Shen et al., 2008). In addition, Al sensitivity was altered by knock-out or over-expression *PIN* genes in rice and Arabidopsis (Sun et al., 2010; Wu et al., 2014, 2015). These results suggest that abnormal PIN-mediated auxin flux in the root apex under Al stress is one of reasons for Al-induced root growth inhibition. Additionally, several Al-inducible IAA synthesizing genes, *AtTAA1* and *AtYUCCA*, encode proteins that regulate IAA accumulation in the root transition zone (TZ) which is located between the root meristem and zone of elongation (Yang et al., 2014; Liu et al., 2016). These genes are specifically induced in the TZ in response to Al, and activate IAA biosynthesis, resulting in root growth inhibition. Lastly, a recent study showed that the multidrug and toxic compound extrusion transporter, DETOXIFICATION 30 (DEX30), regulates auxin homeostasis in the TZ under Al stress, and contributes to Arabidopsis Al tolerance (Upadhyay et al., 2020).

Abscisic acid (ABA) also appears to be involved in Al signaling. Similar to several other phytohormones, endogenous ABA levels are upregulated under Al stress (see, for example, Kasai et al., 1995). However, unlike auxin and ethylene, ABA positively regulates Al tolerance. Al-induced root growth inhibition is alleviated by exogenous ABA application in barley, soybean, and buckwheat (Kasai et al., 1993; Shen et al., 2004; Hou et al., 2010; Reyna-Llorens et al., 2015). Additionally, co-treatment of ABA and Al induce greater root tip organic acid release than Al alone in soybean (Shen et al., 2004). In addition, ABA induces *AtALMT1* and *AtMATE* expression and malate release without Al in Arabidopsis (Kobayashi et al., 2013b). Therefore, Al-induced ABA production may contribute to the activation of OA transporter expression and increased OA release, which leads to Al resistance. Interestingly, IAA also induces *AtALMT1* expression, but it cannot activate malate release from roots without Al. This result is consistent with the finding that IAA treatment does not enhance Arabidopsis Al resistance.

#### Phosphatidylinositol

Recently, Wu et al. (2019) reported that blockade of phosphatidylinositol (PI) signaling, especially the Phosphatidylinositol 4-kinase (PI4K) and phospholipase C (PLC) pathways, leads to down-regulation of a number of Al-inducible genes, including *ALMT1*. PI and its derivatives are membrane lipids and conserved as signaling molecules among eukaryotes, and are involved in various important biological process such as membrane trafficking, root hair and pollen tube tip growth, and stress responses in plants (Meijer and Munnik, 2003; Thole and Nielsen, 2008; Ischebeck et al., 2010; Hou et al., 2016). In the screening, PIK-75 (Inhibitor for phosphatidylinositol 3-kinase [PI3K] in human) was identified that inhibits Al-induced malate secretion due to reduction of *ALMT1* expression. *In silico* docking analysis suggested that PIK-75 can interact with PI3K and PI4K in Arabidopsis. They confirmed that the PI4K and the subsequent PLC pathways play critical roles in Al-inducible *ALMT1* expression. Additionally, the blocking of the PI4K/PLC pathways significantly suppresses several Al-inducible genes, including STOP1-dependent target genes. The PI3K inhibitor does not affect Al-induced gene expression, suggesting that the PI4K/PLC pathways uniquely regulate signaling pathways associated with Al-inducible gene expression. However, PI3K is involved in plant Al signal transduction, because the PI3K inhibitor reduces root malate exudation via activation of the ALMT1 protein. More than 20 years ago, (Jones and Kochian, 1995) already speculated that the relationship between Al toxicity and membrane lipids included phosphatidylinositol. They found that Al directly and strongly binds to several plasma membrane lipids. PI(4,5)P2, the intermediate product between the PI4K and PLC pathways, has highest binding affinity with Al^3+^. In addition, inositol trisphosphate, which is one of final products in the PI4K/PLC pathways, is transiently accumulated in culture coffee cells under Al stress (Poot-Poot and Teresa Hernandez-Sotomayor, 2011). These findings suggest that Al alters PI signaling/metabolism, and this could be a possible sensing mechanism for Al stress in plants.

## P Deficiency Stress and Responses

Root system architecture (RSA) alterations leading to longer and thinner ageotropic lateral roots in the topsoil (where P levels are highest) is essential for the plants to more effectively forage for P in the soil, increasing P acquisition under low soil P availability (Lynch, 2011). The main processes that affect RSA and increase root exploration capacity stem from cell division in the root pericycle ahead of generation of lateral root meristems, which allows for indeterminate growth, and the formation of seminal and lateral roots arising from lateral meristem initials in the pericycle of the root stele (López-Bucio et al., 2003).

Root remodeling in soils with low-P availability is related to two types of signaling pathways. Local signaling is associated with RSA modifications regulated by changes in the rhizosphere P concentration in the soil, with the root apical meristem (RAM) being the site sensing the P changes in the soil (Chien et al., 2018). Under low-P conditions, the differentiation of meristematic and stem cells especially in the pericycle, where lateral roots arise, are triggered (Sánchez-Calderón et al., 2005; López-Bucio et al., 2019; Wang et al., 2019). The second P signaling pathway is systemic signaling, where low soil P availability results in lower shoot P availability, triggering systemic responses transmitted to the root to reprogram root processes enhancing P acquisition. The primary conduit for these systemic responses is the phloem, which in addition to sugars produced by photosynthesis in mature leaves, also contains hundreds or thousands of different RNA species and proteins that can serve as signaling molecules for plant responses.

The best example of this is plant response to P deficiency, which triggers massive changes in the phloem transcriptome and proteome (Zhang et al., 2016; Zhang Z. et al., 2019). The first example of P deficiency systemic signaling involves the *microRNA 399* (*mirR399*), which is induced early in the low-P response in leaves and moves to the root in the phloem to interact with its target, the *PHO2* gene (Fujii et al., 2005; Chiou et al., 2006; Hu et al., 2015). The transcription factor AtMYB2 acts as a direct transcriptional activator of *miR399* (Baek et al., 2013), and *miR399* then can directly cleave *PHOSPHATE 2* (*PHO2*) mRNA in some species (Bari et al., 2006; Ramírez et al., 2013; Ouyang et al., 2016). PHO2 is a ubiquitin-conjugating E2 enzyme (UBC24) that negatively regulates P transporters, inhibiting P uptake and root-to-shoot translocation under sufficient P conditions (Aung et al., 2006; Bari et al., 2006). Subsequent studies showed that PHO2 targets proteins that are involved in expression of the root high affinity uptake transport genes, *Pht1;8* and *Pht1;9*. *miR399* is strongly induced by P deficiency in source leaves and then loaded into the phloem, where it is translocated to the root and silences *PHO2*, which in turn allows high expression of *Pht1;8/Pht1;9* and increased root P uptake (Fujii et al., 2005; Bari et al., 2006; Chiou et al., 2006; Hsieh et al., 2009). In the Zhang et al. (2016) paper cited above, the authors directly identified and quantified mRNAs that move from the shoot toward the root in the phloem, and whose abundance are altered by P deficiency. They used the appearance of *miR399* in the phloem as a bioassay for the plant perceiving P deficiency in the shoot and found it appeared in the lower source leaf phloem rapidly, within 12 h after withholding P from the roots. In this study they found that imposition of Pi stress induced large and rapid changes in the mRNA population in the phloem, and grafting studies demonstrated that many hundreds of phloem-mobile mRNAs are delivered to specific sink tissues, including the root. From these findings the authors proposed that the shoot vascular system acts as the site of perception for root-derived Pi stress signals, and the phloem delivers a cascade of signals to the different plant sinks, in order to coordinate P status throughout the plant. The molecular mechanisms for both local and systemic signaling that orchestrate P sensing and activation of pathways induced by low-P availability are not fully understood. The cross-talk between regulatory networks certainly occurs, but the information available is still fragmented, so this topic will focus on the transcriptional networks and molecules involved in P response and root remodeling.

MicroRNA 399 plays this key role in Pi-starvation signaling network in many plant species other than Arabidopsis. Its rice homolog, *LEAF TIP NECROSIS1* (*LTN1*), is associated with root morphology changes under low-P, and the lack of function *ltn1* mutant exhibits elongation of primary and adventitious roots under P starvation. In rice, *LTN1* is a key component downstream of *miR399* in the P starvation response (Hu et al., 2011). In maize, *miR399* transcripts are strongly induced in maize by P deficiency. Moreover, lines overexpressing *MIR399b* accumulated more P in their shoots, showing P-toxicity phenotypes and presented significantly lower abundance of the long-noncoding RNA1 (*PILNCR1*) in P-efficient lines, indicating that the interaction between *PILNCR1* and *miR399* is important for tolerance to low-P (Du et al., 2018). Finally, the overexpression of the transcription factor, *WRKY74*, in rice led to a larger root system phenotype, enhanced P acquisition and grain yield (Dai et al., 2016). These authors also showed that OsWRKY74 likely is a positive regulator of *miR399*.

### PHOSPHORUS-STARVATION TOLERANCE 1 (PSTOL1) genes

To date, there are not many genes that directly link root morphology and P acquisition, particularly in crop species cultivated in soils with low-P availability. A receptor-like cytoplasmic kinase gene named *PHOSPHORUS-STARVATION TOLERANCE 1* (*PSTOL1*) described by Gamuyao et al. (2012), is the first candidate P efficiency (tolerance to low soil P) gene identified. This gene encodes a receptor-like kinase and is responsible for a major quantitative trait locus for rice root P uptake (Wissuwa et al., 2002). Rice lines overexpressing *PSTOL1* showed greater root total length and root surface area (Gamuyao et al., 2012), and enhanced phosphorus uptake and grain yield under low-P conditions compared to the control. PSTOL1 is expressed in the crown root primordial and parenchymatic cells located outside of the peripheral vascular cylinder, where crown roots are formed in rice (Gamuyao et al., 2012). Although P-starvation induced (*PSI*) genes were not differentially regulated by PSTOL1, constitutive genes with regards to P supply, such as HOX1 (Scarpella et al., 2005), a transcription factor that is a positive regulator of root cell differentiation, was up regulated in lines overexpressing *PSTOL1* in the Gamuyao et al. (2012) study, which is consistent with the proposed role of PSTOL1 in regulating early crown root development and root growth in rice.

In sorghum, multiple homologs of *OsPSTOL1* were shown by candidate gene association mapping to be associated with P efficiency in the field (grain yield and P uptake on low-P soil) and/or in the lab (changes in root topology and growth, and P uptake; Hufnagel et al., 2014). In this study, these sorghum *SbPSTOL1* genes appear to modify root system morphology and architecture, leading to increases in grain yield in field studies on a low-P Brazilian soil, and also exhibited enhanced biomass accumulation and P content in sorghum landraces from West Africa using native soils. These data suggest a stable effect of the target alleles across environments and sorghum genetic backgrounds (Hufnagel et al., 2014; Bernardino et al., 2019). In maize, homologs of *OsPSTOL1* that were preferentially expressed in roots and co-localized with QTLs associated with root morphology and P acquisition traits (Azevedo et al., 2015), mapped in the same region as QTLs for grain yield on a low-P soil (Mendes et al., 2014).

### TFs Involved in Plant Low-P Response/P Efficiency

The maize transcription factor (TF) ROOTLESS CONCERNING CROWN AND SEMINAL ROOTS (RTCS) has been shown to be involved in altering root development and architecture (Hetz et al., 1996; Taramino et al., 2007). More recently, Salvi et al. (2016) also reported co-mapping of a quantitative trait loci controlling the number of seminal roots in maize, with the *RTCS* gene. RTCS contains a Lateral Organ Boundaries (LOB) domain, LBD, that is induced by auxin. RTCS acts downstream of ARF34 (and is responsible for the initiation of embryonic seminal and postembryonic shoot-borne roots (Xu et al., 2015). Other RTCS LBD proteins are involved in several developmental processes; for example, Arabidopsis LBD16/ASYMMETRIC LEAVES18 (ASL18) is involved in the regulation of lateral root formation, downstream of ARF7 and ARF19 TF’s (Lee et al., 2009). RTCS was highly expressed in a P efficient maize genotype under low-P conditions when compared to a P inefficient genotype (De Sousa et al., 2012), indicating that is modulated by maize P status.

The TF *PTF1* (*phosphorus starvation transcription factor*) is a member of the *BASIC HELIX-LOOP-HELIX* (*bHLH*) family of TF’s and plays a role in low-Pi tolerance response in rice, maize and soybean (Yi et al., 2005; Li et al., 2011; Li Z. et al., 2019). In maize, ZmPTF1 is involved in the promotion of lateral root development and also binds to the promoter and positively regulates the transcription of a number of other TF’s including *9-cis-epoxycarotenoid dioxygenase (NCED)*, *C-repeat-binding factor (CBF4)*, *ATAF2/NAC081*, and *NAC30*. RNA-seq data showed that genes related to the auxin signaling pathway are also up-regulated in *ZmPTF1* overexpression lines (Li Z. et al., 2019). These authors suggested that ZmPTF1 acts upstream of signaling pathways related to biosynthesis and activation of phytohormones such as ABA and auxin, which are associated with root system development and the Pi starvation and drought tolerance responses.

There are a number of other WRKY TFs involved in P deficiency stress. One of these is WRKY6, which negatively regulates *PHO1* expression under normal, sufficient P conditions. PHO1 is the phosphate efflux transporter that mediates xylem loading of Pi in the roots. When the plant experiences P deficiency, WRKY6 is degraded via 26S proteasome-mediated proteolysis (Chen et al., 2009). Its homolog in Arabidopsis, WRKY42, also negatively regulates *PHO1* transcription under P sufficiency, but under the same plant P status, it positively regulates expression of the gene encoding the root Pi uptake transporter, PHT1;1 (Chen et al., 2009; Su et al., 2015). Under P deficiency, like WRKY6, WRKY42 is also degraded by the 26S proteasome. Another related TF, *WRKY45*, whose expression is root-specific, binds to two W box elements in the promoter of *PHT1* and regulates its transcription (Wang et al., 2014). WRKY75 appears to play dual roles in P deficiency responses. It is an activator of expression of a number of P deficiency induced genes, including phosphatases and P transporters (Devaiah et al., 2007). But it also is a negative regulator of root development associated with P deficiency. That is, when it is knocked out, lateral root length and number, and root hair density, were significantly increased. Hence, *WRKY75* is the first WRKY transcription factor to be shown to regulate both a nutrient deficiency response and root development and architecture.

### Major Al Tolerance Genes That Are Also Involved in P Deficiency Stress Pathways

As plants that have adapted to highly acidic soils have had to deal with the dual stresses of Al toxicity and low soil P availability/high P fixation (Kochian et al., 2015), it is not surprising researchers have recently begun to discover that what were believed to solely be Al tolerance genes also can be involved in P deficiency responses and possibly P efficiency. These findings come from research on Arabidopsis, and the three key players in this scenario are STOP1, the TF that regulates Al-induced expression of a number of Al tolerance genes (Iuchi et al., 2007), and two of the genes regulated by STOP. These are *ALMT1*, the root tip PM malate anion channel that is activated by Al and releases Al chelating malate into the acid soil rhizosphere (Hoekenga et al., 2006), and *ALS3*, whose function is more varied and puzzling. ALS3 was first shown by Larsen et al. (2005) to be an Al tolerance gene that encodes an ABC transporter that in the shoots, is localized to the vasculature and hydathodes. The authors showed in the shoot it was PM-localized and speculated it could confer Al tolerance by loading Al into the phloem, thus moving it away from the site of toxicity in the root tip.

More recently, Dong et al. (2017) found that in Arabidopsis roots, knockout of *ALS3* results in hypersensitivity to low-P. In this study, ALS3 was found to be part of a root tonoplast ABC transporter complex with AtSTAR1, which is the counterpart of rice OsSTAR1, which in rice pairs with OsSTAR2 (the rice counterpart of ALS3) to form a cytoplasmic vesicle ABC transporter involved in rice Al tolerance (Huang et al., 2009). The Arabidopsis ALS3/AtSTAR1 transporter complex was shown to mediate electrogenic transport in oocytes (transports net charge across the membrane), but it is not clear what solute AtSTAR1/ALS3 transports across the root-cell tonoplast. This study is one of several (the others being; Müller et al., 2015; Balzergue et al., 2017; Mora-Macías et al., 2017) that together explain the primary Arabidopsis P deficiency response, which is inhibition of primary root growth and continued lateral root growth under low-P growth conditions. This response involves the genes initially shown to be involved in Al tolerance, *ALS3*, *ALMT1* and *STOP1*. The low-P inhibition of primary root growth requires Fe to occur, and under low-P conditions, Fe accumulation both into the root symplasm and the cell wall is increased. The path of events that start with P deficiency under sufficient/high Fe growth conditions and end with inhibition of Arabidopsis primary root growth are both elegant and relatively complex. These events are summarized here:

(1) P deficiency inhibition of Arabidopsis primary root growth requires available Fe in order to occur.
(2) Under P deficiency, STOP1 induces *ALMT1* gene expression; subsequently the ALMT1 protein releases malate into the root tip apoplast and rhizosphere where it increases Fe availability in the apoplast via chelation of Fe^3+^ from the rhizosphere.
(3) At the same time, P deficiency induces the release of the multicopper ferroxidase, LPR1, from the ER to the cell wall of RAM cells surrounding stem cells in the RAM. LPR1-mediated reduction/oxidation of ferric/ferrous ions in this cell wall region generates peroxide, which catalyzes lignification and cell wall stiffening, accounting for the initial rapid inhibition of root growth.
(4) Concurrently, the ROS generation from LPR1-mediated ferroxidase activity triggers callose formation in this region of the RAM, which blocks plasmodesmata between the stem cells and cells surrounding the stem cell niche.
(5) This prevents for cell-to-cell transport of the TF, SHORT-ROOT, which is essential for stem cell division. This inhibition of stem cell division exhausts the meristem, resulting in the slower inhibition and termination of primary root growth.

The way that cells in the RAM perceive P deficiency is not understood, however, it is known that the accumulation of AtSTOP1 in the nucleus is the on-off switch for the regulatory mechanisms involved in the inhibition of primary root growth associated with P deficiency and Fe accumulation. Wang et al. (2019), building upon the research presented in Dong et al. (2017), showed that STOP1, ALMT1, and LPR1 act downstream of ALS3/STAR1 in controlling Arabidopsis primary root growth in response to P deficiency. Furthermore, they found that the tonoplast ABC transporter, ALS3/STAR1, represses STOP1 protein accumulation in the nucleus, thus inhibiting *ALMT1* transcriptional activation. They suggested that an unknown metabolite or ion is sequestered in the vacuole by ALS3/AtSTAR1, and this metabolite or ion is necessary for STOP1 accumulation in the nucleus. Subsequently, Godon et al. (2019) found that the stability of AtSTOP1 in the nucleus is triggered by Fe^3+^ accumulated in root cells under P deficiency, and not the decrease in P itself. They also found that Al^3+^ had the same effect as Fe on stimulating STOP1 accumulation in the nucleus, which is consistent with the abundance of toxic Al^3+^ ions in acidic soils. The authors suggested that the AtALS3/AtSTAR1 transporter may be mediating the accumulation of either ionic Fe or Al, or Fe/Al chelates in the vacuole, and in the case of P deficiency, this transporter controls cytoplasmic Fe homeostasis via the stability of AtSTOP1 in the nucleus under low-P conditions.

### Involvement of Posttranslational Modification in P Deficiency Responses

SUMO E3 LIGASE (SIZ1) is responsible for post-translational modifications based on the addition of small Ubiquitin-like Modifier (or SUMO) proteins, which can affect protein function (Gareau and Lima, 2010). The MYB-like transcription factor, PHOSPHATE STARVATION RESPONSE1 (PHR1), which modulates RSA under P starvation, is one example of a protein modified by sumoylation (Miura et al., 2011). In rice, OsMYB2P-1 positively regulates P starvation signaling and lines overexpressing this gene have a longer primary root and more lateral roots compared to the wild type under low-P conditions (Dai et al., 2012). PHR1 and its homolog PHL1 (PHR1-Like1) directly bind to the *cis*-element, P1BS (Rubio et al., 2001), which is prevalent in the promoters of many P starvation induced genes, including *PHO1*, *miR399*, *IPS1* (*INDUCED BY PHOSPHATE STARVATION1*), and *RNS1* (*RIBONUCLEASE1*) (Poirier et al., 1991; Bariola et al., 1994; Martín et al., 2000; Bari et al., 2006). PHR1 has also been found to be sequestered from the nucleus in a P-dependent manner by SPX1, a nucleus-localized SYG/PHO81/XPR1 domain protein, inhibiting PHR1 activity (Puga et al., 2014). In rice, SPX4 negatively regulates *PHR2*; under low-P, *SPX4* degradation is accelerated through the 26S proteasome pathway, releasing PHR2 into the nucleus and activating the expression of *PSI* genes (Lv et al., 2014). Getting back to sumoylation, a loss-of-function *siz1* mutant exhibited reduced primary root growth and increased lateral root and root hair length and density, which is apparently independent from the PHR1/SIZ1 signaling pathway (Miura et al., 2011). SIZ1 is also involved in the negative regulation of auxin patterning to modulate RSA in response to low-P (Miura et al., 2011).

This *siz1* mutation also revealed a dual role of the SIZ1 E3 ligase in the regulation of P homeostasis in rice. In *siz1* rice plants grown under P deficiency, two root high-affinity P transporter genes, *OsPT1* and *OsPT8*, were more highly expressed compared to the WT, whereas *OsPT2* and *OsPT6* (which are expressed in both roots and shoots) were down-regulated (Wang et al., 2015). OsPT2 and OsPT8 are phosphorylated by CASEIN KINASE2 (CK2), which inhibits their interaction with PHOSPHATE TRANSPORTER TRAFFIC FACILITATOR1 (OsPHF1) under normal conditions. OsPHF1 is a SEC protein that facilitates the trafficking of Pi transporters from the ER to the PM (González et al., 2005). The retained phosphorylated phosphate transporters in the endoplasmic reticulum lead to a reduced P absorption from the rhizosphere (Chen et al., 2015). Also, rice PROTEIN PHOSPHATASE95 (OsPP95), a PP2C protein phosphatase negatively regulated by OsPHO2, positively regulates P homeostasis and remobilization, through the interaction with OsPT2 and OsPT8. OsPP95 acts antagonistically with CK2 to regulate the reversible phosphorylation of phosphate transporters (Yang et al., 2020b).

Another transcriptional factor with a role in P homeostasis is WRKY6, which was shown to negatively regulate the expression of *PHO1* (Chen et al., 2009), which is a phosphate efflux transporter localized to the Arabidopsis root vasculature and is key in loading Pi absorbed by roots from the soil into the xylem for translocation to the shoot (Hamburger et al., 2002). Its closest Arabidopsis homolog, WRKY42, also negatively regulates *PHO1* transcription under P starvation, (Chen et al., 2009; Su et al., 2015). Interestingly, under Pi-sufficient conditions, WRKY42 positively regulates *PHT1;1* expression, which is a root high and low affinity Pi uptake transporter in Arabidopsis (Shin et al., 2004). WRKY42 accomplishes this by binding directly to the *PHT1;1* promoter, and this binding is abolished by low-Pi stress. During Pi starvation, the WRKY42 protein is degraded through the 26S proteasome pathway. These results show that AtWRKY42 modulates Pi homeostasis by regulating the expression of *PHO1* and *PHT1;1* to adapt to environmental changes in Pi availability.

### Members of the Proteaceae Family Have Evolved Unique Adaptations to Acquire P From Low-P Soils

Some plant species of the Proteaceae family develop cluster or proteoid roots in response to growth on low-P soils. Cluster roots are specialized primary lateral roots that develop one or more clusters of rootlets along their axes. Cluster roots synthesize large amounts of organic acid, such as citrate and malate, which are subsequently released into the rhizosphere to increase P availability by chelating metals such as Fe, Al, and Ca that are fixing the phosphate anions in the soil (Keerthisinghe et al., 1998; Neumann et al., 2000; Peñaloza et al., 2002). A number of genes are involved in the developmental and biochemical responses in cluster roots. These include upregulation of the root high-affinity phosphate transporters, LaPT1, and phosphoenolpyruvate carboxylase 3 (LaPEPC3) under P deficiency. Also, it was found that white lupine homologs of the Arabidopsis SCARECROW (AtSCR), LaSCR and LaSCR1 are localized to the root endodermis and presumably help drive the developmental processes that result in these impressive clusters of laterals, which play such an important role in lupine adaptation to low-P soils (Peñaloza et al., 2005; Sbabou et al., 2010).

Recently, a cultivated accession of white lupin was sequenced and de novo assemblies of a landrace and a wild relative were also performed (Hufnagel et al., 2020). The modern accession displays an increased soil exploration capacity through early establishment of lateral and cluster roots (Hufnagel et al., 2020). The authors identified the presence of AP2/EREBP, a large multigene family that is key to control of lateral root development. They also identified several mature microRNAs expressed in cluster root sections and related to P deficiency responses, such as *miRNA156*, *miRNA166*, *miRNA211139*, and members of *miRNA399* family, that were not detected previously in white lupin. Moreover, Hufnagel et al. (2020) identified five genes that are targets of the detected miRNAs, including the TFs *LaWRKY* (*Lalb_Chr07g0182001*) and *LaPUCHI*-3 (*Lalb_Chr18g0055601*). Activation of key regulatory genes may trigger the early establishment of the root system, and consequently P-uptake and P efficiency (increased grain yield on low-P soils).

## Drought Stress and Tolerance

Drought stress is the most widespread abiotic stress affecting crop yield and quality. Due to the sessile nature of plants, evolutionary adaptations have enabled plants to develop sophisticated mechanisms to tolerate or avoid drought. When plants sense water deficit in the surrounding environment, it leads to the generation of drought stress signals (Blackman and Davies, 1985; Kuromori et al., 2014; Batool et al., 2019). These primary and secondary drought response signals are perceived by receptor molecules which leads to direct changes in the expression of genes or expression of TFs that regulate expression drought-responsive genes, which ultimately leads to drought adaptation (Kuromori et al., 2014). Drought signaling networks are presumably complex and to date poorly understood, but it is clear they involve both intercellular and intracellular signaling (Kuromori et al., 2014). Because this review focuses on root adaption to multiple stresses, here we will focus on the role of drought-related communication between the roots and shoots involving intercellular signaling networks and TFs responsive to drought.

### Drought Signaling Molecules

#### Hormones

Several studies have shown that phytohormones act as signaling molecules in response to drought. ABA is one of the most widely studied phytohormones in part because of its role in regulating stomatal conductance in response to different related abiotic stresses that impact plant water status including drought, salinity, high and low temperatures. Jones and Mansfield (1970) showed that external application of ABA to roots led to a reduction in stomatal aperture suggesting that ABA was involved in regulation of stomatal conductance. This led a number of researchers to conduct plant water stress studies investigating the hypothesis that root-derived ABA is a primary candidate for root to shoot drought signaling. It had been generally accepted that stomatal closure in response to drought was triggered by reductions in leaf water potential due to the drought conditions. A key finding in changing thinking about drought signaling came from the work of Blackman and Davies (1985), which showed that reduced water content in roots in response to drought led to stomatal closing or reduction in stomatal aperture without changes in leaf water potential. This indicated that a signal was likely traveling from the root to leaves to help induce stomatal closure. As described above regarding the earlier work of Jones and Mansfield (1970), ABA was already known to decrease stomatal opening and thus it became a logical candidate for a drought-induced root signal transmitted to leaves. Subsequently, it was shown that upon soil drying, the ABA concentration was increased in maize roots and xylem sap (Zhang and Davies, 1989, 1990a), and these findings further strengthened the idea of ABA as a key drought induced signal in root to shoot signaling. Subsequent work with a number of plant species, including maize, sycamore, lupin, wheat, castor bean and grapevine (Loveys and Kriedemann, 1974; Zhang et al., 1987; Henson et al., 1989; Zhang and Davies, 1990b) all showed that soil water deficit induced ABA synthesis in roots, and the newly synthesized ABA was then translocated to leaves via the xylem to induce stomatal closure.

Drought stress can cause arrested shoot growth; however, it has been shown that under those conditions root elongation can continue due to ABA-mediated plant adaptations (Sharp and Davies, 1989; Saab et al., 1990; Sharp et al., 1994) observed that primary root elongation was maintained under water limited conditions, and in subsequent work it was suggested that increased ABA accumulation in the root under drought conditions (water potential [ψ_w_] of −1.6 MPa) might play a key role in the prevention of root growth elongation inhibition under drought stress (Saab et al., 1990). In the Saab et al. (1990) study, two treatments were employed. These involved both the root application of fluridone, an inhibitor of carotenoid biosynthesis that provides the precursors for ABA biosynthesis, and the use of the *vp5* mutant that is deficient in carotenoids that leads to reduced ABA synthesis. They showed that in both of these treatments, the roots did not maintain continued root elongation at a lower water potential compared to wild type maize plants. They also conducted these experiments in a dark environment with saturated humidity to rule out the indirect effects of ABA deficiency on photosynthesis and/or alterations in stomatal control. Subsequently, Sharp et al. (1994) confirmed the role of ABA in maintenance of tap root growth under water limited conditions by applying exogenous ABA, which recovered the wild type root growth phenotype in both the *vp5* mutant and fluridone-treated plants under drought. Based on an earlier report by Wright (1980) on the interaction between ABA and ethylene, researchers from the Sharp lab also investigated whether ABA-dependent inhibition of ethylene synthesis was involved in the maintenance of root elongation under water limited conditions (Spollen et al., 2000). In this study, wild type root growth elongation was recovered by applying ethylene synthesis and action inhibitors in the vp5 mutant and in fluridone-treated maize plants, suggesting that the ability of root growth to better tolerate drought compared to shoot growth does involve interactions between ABA and ethylene.

Despite this body of work supporting the hypothesis of root to shoot translocation of ABA in response to drought, other studies suggesting the importance of leaf ABA synthesis have been carried out in a number of labs using reciprocal grafting between wild type and ABA deficient mutants in tomato, Arabidopsis, and sunflower. In these studies, drought stress was imposed on the wild type shoot/mutant roots and mutant shoots/wild type roots grafting combinations. When these different grafted “genotypes” were water stressed, the shoot genotype was shown to control stomatal behavior, suggesting that shoot-derived ABA was also important drought response (Jones et al., 1987; Fambrini et al., 1995; Holbrook et al., 2002; Christmann et al., 2007; Dodd et al., 2009). In summary, despite the general acceptance in the plant water relations that the primary mode of ABA signaling occurs via root ABA synthesis, followed by translocation via xylem to the leaves, it is clear that the field of plant ABA signaling is not unified behind this hypothesis. As supported by the findings reported in the publications summarized in this section of the review, ABA signaling may involve both roots and leaves, with a systemic response involving ABA that is made in roots under drought and transported to leaves, and a more local response within the drought-stressed leaf.

The ABA signaling pathway has been studied extensively in the model plant, *Arabidopsis thaliana*. ABA receptors have been identified in Arabidopsis, and during ABA signaling, ABA has been shown to bind to intracellular ABA receptors from the PYR/PYL/RCAR family, triggering a signal cascade that results in ABA-mediated stomatal closure (Fujii et al., 2009; Nishimura et al., 2009; Santiago et al., 2009; Cutler et al., 2010; Gonzalez-Guzman et al., 2012). The binding of ABA to its receptor leads to interactions with a Type 2C protein phosphatase (PP2C), which inhibits PP2C-mediated activation of an OST (Open stomata) 1 kinase. This kinase is responsible for stomatal opening by controlling anion channels in the guard cell plasma membrane, and blocking its activation results in stomatal closure (Ma et al., 2009; Park et al., 2009; Hauser et al., 2017). Hence, PP2C is a negative regulator of the ABA signaling pathway, resulting in stomatal closure. Interesting recent findings from Belda-Palazon et al. (2018) showed that the PYL8 ABA receptor is responsible for root perception of ABA though a non-cell-autonomous mechanism. In this study the PYL8 transcript was localized to the root meristem epidermis and stele, while the PYL8 protein was also found in adjacent tissues. The authors go on to show that both inter- and intracellular trafficking of PYL8 appears to occur in the RAM. This study shows that ABA receptors can interact with ABA in the root. It doesn’t appear that this interaction plays a role in drought signaling to the shoot. Instead the authors hypothesize that the binding of ABA to the PYL8 receptor in the root may be involved in well documented roles of ABA in root function including root growth associated with hydrotropism and salt stress, and root plasticity in response to variation in nutrient availability (Barberon et al., 2016; Feng et al., 2016; Dietrich et al., 2017).

There are a number of published papers indicating that ABA biosynthesis occurs in both shoots and roots, and this occurs first via biosynthetic processes in plastids, and then the ABA precursors made in the plastid are transported to the cytosol, where they are converted to ABA (Thompson et al., 2007; Fujii et al., 2009; Nishimura et al., 2009; Santiago et al., 2009; Cutler et al., 2010). With regards to drought signaling regulation of stomatal function, cytosolic ABA has been found to bind to PYR1-type ABA receptors also located in the cytoplasm (Fujii et al., 2009; Nishimura et al., 2009; Santiago et al., 2009; Cutler et al., 2010). Based on the findings presented above for (Belda-Palazon et al., 2018), it is clear that ABA receptors are both functioning in the root and the leaf. In the studies showing that ABA binds to PYR1-type ABA receptors in leaf tissue, although not directly stated, the clear implication is that this ABA interaction with its receptor occurs in the guard cell cytoplasm, although that has not yet been shown.

Components of the ABA signaling pathway that are involved in moving ABA either into guard cells or from roots to leaves have been found. Kuromori et al. (2010) identified an ABC (ATP binding cassette) efflux transporter gene *AtABCG25* that encodes an ABC transport protein localized in the root vascular parenchyma plasma membrane. This transporter exports ABA accumulated in root xylem parenchyma cells into xylem vessels in response to drought stress. Studies also showed that transgenic Arabidopsis plants overexpressing *AtABCG25* had higher leaf temperatures and lower transpirational loss of water from detached leaves, compared to wild type plants. This is consistent with more ABA being provided to guard cells from the root, leading to stomatal closure. Furthermore, Kang et al. (2010) has identified an ABA uptake transporter, AtABCG40 (also known as Pleiotropic drug resistance transporter PDR12). This ABC transporter is localized to the plasma membrane and predominantly expressed in leaf guard cells, where it acts to transport ABA that is delivered via the xylem, into guard cells. In summary, many of the structural components of the root to shoot ABA signaling network are being identified, including root-localized ABA biosynthetic pathways, transporters involved in xylem translocation of ABA to the shoot, and transporters in the leaf moving ABA into guard cells.

Another hormone that appears to be involved in drought signaling is cytokinin. Reduced maize stomatal aperture due to exposure to dry soil was reversed by the application of cytokinin to the roots (Blackman and Davies, 1985). Whereas xylem levels of ABA are increased in response to drought treatment of rice seedlings, cytokinin levels are decreased under the same drought conditions (Bano et al., 1993). These findings suggest that both hormones are involved in drought signaling, acting antagonistically to more finely regulate stomatal aperture related to plant water status (Blackman and Davies, 1985; Bano et al., 1993).

#### Peptide Hormones

The CLE (CLAVATA3/EMBRYO-SURROUNDING REGION) family of peptides are small peptides that function as plant hormones via release from cells into the extracellular space, where they function as intercellular signaling molecules. They have been shown to bind to receptor-like proteins at the outer surface of the plant cell plasma membrane to help mediate signal transmission. CLE peptides have been shown to regulate a range of physiological and developmental processes, including drought responses. CLE proteins have recently been shown to be a mobile signal transmitted from roots to shoots and involved in increased ABA biosynthesis after dehydration stress (Takahashi et al., 2018). In this study, synthetic isotope-labeled CLE25 was externally applied to roots and its accumulation was detected in leaves of treated plants using nanoscale nLC-MS/MS. The CLE25 peptide has been shown to be involved in regulation of ABA biosynthesis in leaves after the roots sense drought conditions in the soil (Takahashi et al., 2018). CLE25 is expressed in vascular tissues and its expression is induced in response to drought. Subsequently, the CLE25 peptide moves from the roots to leaves, where it enhances ABA synthesis and accumulation, helping trigger stomatal closure. It does this by binding to BARELY ANY MERISTEM (BAM) receptors in leaves. It is possible that CLE25 plays a role in the leaf-mediated regulation of stomatal function described above from the earlier publications reporting on the physiology of drought induced stomatal closure. If this is the case, then CLE2 could be a second root-to-shoot drought signal (the other being ABA itself) that triggers leaf-localized ABA regulation of stomatal responses to drought.

#### Other Signals

Plant cellular and apoplastic pH has been proposed to be another signaling factor that could play a role in stomatal aperture regulation (Hartung et al., 1998; Wilkinson, 1999). Drought stress has been shown to trigger an increase in xylem sap pH (Gollan et al., 1992; Wilkinson and Davies, 1997; Hartung et al., 1998). Under these conditions, Wilkinson and Davies (1997) found that this led to an increase in apoplastic ABA in the leaves. They hypothesized that as drought increases ABA concentrations in the xylem sap, and are then transported to the leaves, the higher apoplastic (xylem) pH will deprotonate acid groups in the ABA molecules and the increased charge of the ABA anion will reduce passive ABA flux through the lipid bilayer of the leaf cell plasma membrane. Hence, they speculated that extracellular ABA may be important in triggering stomatal closure. This is a topic that will require more research to more clearly define both the role of xylem pH in drought signaling and the role of apoplastic ABA in directly regulating stomatal response to drought.

Recent studies have identified specific microRNAs that are responsive to drought stress (Bakshi and Oelmüller, 2014; Bakhshi et al., 2016; Aravind et al., 2017) and this could be part of another drought signaling mechanism, as microRNAs can regulate genes post-transcriptionally (Aukerman and Sakai, 2003). Bakhshi et al. (2016) identified 61 known and 11 novel microRNAs involved in drought signaling in rice by performing experiments with a divided root system where half of the root system was water stressed and the other half kept well-watered. They identified miRNAs that exhibited differential expression when the entire root system is exposed to drought stress, along with miRNAs whose expression was altered in response to divided root system drought versus well-watered signaling. The results for differential expression of many of the miRNAs were validated via qRT-PCR. Furthermore, *in silico* target analysis led to the identification of two to three hundred novel target genes for the drought stress response of the entire root system, along with responses of the divided root system to drought and well-watered conditions. From the target analysis, the authors proposed these miRNAs could be involved in a number of drought response pathways, including ABA and calcium signaling, detoxification of free radicals induced by drought, and stimulation of lateral root initiation and growth, which could lead to bigger and deeper root systems that could more effectively acquire water located deeper in the soil profile under drought.

### TFs Responsive to Drought

It is well known that transcription factor (TF) proteins can play major roles in regulatory and signaling networks, and plant drought response is no exception. A number of recent studies have been conducted to identify TFs responsive to drought and in some these studies, the function of the identified TFs has been elucidated (He et al., 2016; Lee et al., 2016, 2017; Chen Y. et al., 2018; Kumar et al., 2019). TFs responsive to drought are members of several different TF families including: (1) the AREB/ABF (ABA responsive binding or ABRE binding factor) family; (2) the AP2/ERF (ethylene response element binding factor) family; (3) the bZIP (the basic leucine zipper) family; (4) the NAC (NAM, ATAF1,2, CUC2) family; and (5) the WRKY transcription factor family (Joshi et al., 2016).

With regards to a TF in the AREB/ABF family involved in drought signaling, Marinho et al. (2016) showed that soybean transgenic lines overexpressing *AtAREB1* exhibited enhanced performance under drought without any penalty on yield. From changes in expression profiles for phosphatases (PP2C) and kinases (SnRK2) in the *AtAREB1*-overexpressing transgenic plants, the authors noted that the observed lower expression of phosphatases and higher expression of kinases are known to be linked to ABA-dependent stomatal closure, and the resulting reduced stomatal conductance to water in the OE lines could explain the observed drought resistance. This overexpression line also had a higher leaf area index and elevated intrinsic water use in subsequent research by Fuganti-Pagliarini et al. (2017). In rice, overexpression of *OsERF71* altered expression of genes that regulate lignin biosynthesis and cell wall loosening enzymes, leading to increased root radial growth, more cell layers in the vasculature, and increased root aerenchyma, and these root structural changes were associated with reduced water transpiration and increased drought tolerance (Lee et al., 2016). OsERF71 belongs to the AP2/ERF TF family and is mainly expressed in the root endodermis, meristem and pericycle tissues. It was not clear how these root structural changes confer drought resistance, but the authors noted that increased radial growth has been observed in other studies in response to drought. The authors pointed out that in these previous studies, it has been suggested that observed increases in aerenchyma could reduce the carbon cost required to produce bigger root systems (Zhu et al., 2010). In the *OsERF71* overexpressing lines, the putative lignin biosynthesis genes, cinnamoyl-coenzyme was expressed ten-fold higher than in wild type plants. This was associated with quantification of higher lignin accumulation in roots tissues by phloroglucinol staining in the transgenic plants. Increased lignin biosynthesis might be required for additional root layer formation for wider radial root growth to accommodate larger aerenchyma.

NAC TFs have been characterized in transgenic wheat and it was reported that *TaRNAC1*-overexpressing lines exhibited changes in root growth and structure, which resulted in larger and deeper root systems and increased performance under drought, presumably due to enhanced water acquisition (Chen D. et al., 2018). Finally, He et al. (2016) evaluated Arabidopsis transgenic lines overexpressing the wheat *TaWRKY33* transcription factor for drought tolerance. They observed that *TaWRKY33* overexpression was associated with increased expression of *ABI5*, which encodes a basic leucine zipper transcription factor that in involved in the regulation of seed germination and early seedling growth under abiotic stress conditions that involve ABA. It has been shown that ABI5 is involved in the receptor-mediated ABA signaling described above (PYR/PYL/RCAR ABA receptors, PP2C phosphatases and SnRK2 kinases), through its interaction with ABSCISIC ACID RESPONSE ELEMENT (ABRE) motifs in target gene promoters. Hence, ABI5 has been shown or proposed to be involved in many ABA-related activities, including seed germination, seedling stress tolerance, integration of hormone interactions, and ABA biosynthesis (for a review, Skubacz et al., 2016).

In the He et al. (2016) publication, the authors suggested that the TaWRKY-mediated increase in *ABI5* expression was likely central to the observed improved performance under drought, possibly due to increased ABA synthesis under drought conditions. In the OE lines they also observed reduced transpirational loss of water from excised leaves, which would correlate with increased ABA accumulation resulting in greater stomatal closure.

### ERECTA- A Leucine Rich Repeat Receptor-Like Kinase

The *ERECTA* gene has been shown to be involved in the regulation of leaf transpirational water loss through stomata by altering leaf anatomy (Masle et al., 2005). Leaf carbon isotope discrimination, which is due to the discrimination against the naturally occurring carbon isotope, ^13^C, in favor of the more abundant ^12^C isotope during photosynthetic CO_2_ fixation by the rate-limiting enzyme, Rubisco, is negatively related to leaf transpiration efficiency (ratio between transpirational water loss and photosynthetic CO_2_ assimilation). Hence, leaf isotope C discrimination can be used as a proxy phenotype for transpirational efficiency. Using this approach, leaf isotope C discrimination was used to phenotype an Arabidopsis Col-4 x Ler RIL population. Genetic analysis of the data yielded a significant QTL for transpirational efficiency for leaf isotope C discrimination that was then fine mapped on chromosome 2 (48.96–51.02 cM) and explained up to 64% of the phenotypic variation for this trait in the RIL population. The population parents, Col and Ler, contain *ERECTA (ER*) and *er1* alleles, respectively. Upon screening of candidate genes residing in that region, they found that the *ERECTA* gene was located in the center of the QTL interval. They also observed contrasting values of leaf isotope C discrimination for individuals with the *ER* or *er1* alleles. For functional validation of the candidate gene for transpirational efficiency, multiple ERECTA mutants were compared with near-isogenic lines containing *ERECTA* allele homozygotes. They observed that all of the *er* mutants exhibited higher leaf isotope C discrimination and therefore lower transpirational efficiency than lines homozygous for *ERECTA* allele. Further, in transgenic lines which complemented the mutation with the *ERECTA* allele, they confirmed the identity of *ERECTA* as a transpirational efficiency gene. By dissecting leaf anatomical features, lower stomatal conductance because of lower stomatal density caused by epidermal cell expansion, was observed as the anatomical effect of the *ERECTA* gene. Loosely packed fewer and smaller mesophyll cells were also observed, and it was concluded that all of these phenotypes collectively are affecting transpirational efficiency. These anatomical phenotypic traits were maintained under drought stress which suggests that the *ERECTA* gene could be an important genetic tool to increase transpirational efficiency in crops in drought stress environments.

Zheng et al. (2015) also showed that the expression of two wheat *TaER* genes were positively correlated with transpiration efficiency and yield traits. Gene sequences for ERECTA orthologs in wheat were identified by Linzhou et al. (2013) using a homology-based cloning approach. Zheng et al. (2015) subsequently found the physical chromosomal location of these genes on chromosomes 6 and 7 by using *in silico* approaches to compare *TaER* cDNA sequences to a wheat genome sequence database. The authors also observed significant variation in expression of these genes among 48 wheat varieties in the flag leaves at grain filling and at the heading growth stages. There was a significant positive correlation in TaER expression with water use efficiency, flag leaf area and yield traits (biomass and gain yield), whereas the rate of transpiration, stomatal density and rate of photosynthesis were negatively correlated. These results were consistent with Masle et al. (2005) and further strengthened the role of ER genes in regulation of transpiration efficiency. In addition to the above studies, Li H. et al. (2019) also showed increased drought resistance in Arabidopsis and maize plants by overexpressing the sorghum *ER* (*SbER2-1*) gene and the transgenic overexpression lines exhibited increased rates of photosynthesis and water use efficiency.

## Possible Common Determinants of Al Tolerance, P Efficiency and Drought Tolerance

### C2H2 TFs

Water deficit may disrupt the lipid bilayer in cell membranes, triggering protein denaturation and accumulation of cellular electrolytes, which may lead to osmotic imbalance in plant cells (Fernando and Schroeder, 2016). Hence, osmotic adjustments play a role in plant adaptation to dehydration via turgor maintenance and by the production of osmoprotectants that maintain proper cellular function (Blum, 2017). Cys2/His2-type (C2H2), zinc fingers are known to play a role in plant abiotic stresses tolerance and emerge as a possible hub controlling tolerance to Al toxicity, low-P and also drought stress. Possible mechanisms whereby C2H2 zinc fingers influence drought tolerance have been recently reviewed by Han et al. (2020). Those mechanisms involve the biosynthesis of solutes in the cell leading to osmotic adjustments, reactive oxygen species scavenging via enhanced antioxidant enzyme activity and ABA-dependent signaling pathways.

As previously described, there is evidence linking the C2H2 transcription factor, STOP1, to both Al tolerance and P deficiency tolerance (see section “Major Al Tolerance Genes That Are Also Involved in P Deficiency Stress Pathways”). This emerging pleiotropic role of STOP1 in abiotic stress tolerance has been further supported by the recent finding that *stop1* knockout lines showed enhanced drought tolerance in Arabidopsis (Sadhukhan et al., 2019). Among the genes suppressed in the *stop1* mutant was the *CBL-interacting protein kinase 23* (*CIPK23*), which may be involved in K^+^/Na^+^ homeostasis via regulation of K^+^ transporters. In agreement with K^+^ involvement in stomatal opening (Munemasa et al., 2015), further complementation experiments suggested that the STOP1 function in drought tolerance occurs via ABA-mediated stomatal closure elicited by CIPK23. A protein phosphatase 2C-family protein, PP2C61, was also repressed in the *stop1* mutant, which provides further indication of ABA-dependency for STOP1. This scenario points toward a highly pleiotropic nature of the transcription factor *STOP1*. In this context, STOP1 enhances *AtMATE*- and *AtALMT1*-mediated Al tolerance (see section “Transcriptional Regulation Involved in Al Tolerance”), inhibits primary root growth and enhances lateral root proliferation under P deficiency, possibly favoring P acquisition via Fe-mediated RAM exhaustion (see section “Major Al Tolerance Genes That Are Also Involved in P Deficiency Stress Pathways”). In addition, STOP1 may also influence both salt and drought tolerance (Sadhukhan et al., 2019). However, STOP1 was suggested to negatively impact drought tolerance in Arabidopsis (Sadhukhan et al., 2019), which may conflict with a possible general role of STOP1 in crop adaptation to acidic, tropical soils, where Al toxicity, P deficiency and drought stress usually co-exist.

### NAC and bHLH Transcription Factors

There are many reports linking NAC TFs including NAM, ATAF, and CUC TFs with drought tolerance (Nakashima et al., 2012), which largely involves ABA-dependent pathways. However, some NAC TFs show very early responses to ABA treatment, probably before endogenous ABA accumulates (Tran et al., 2004). Hence, some NACs are also thought to function through ABA-independent pathways (Singh and Laxmi, 2015), at least to some extent. Mutant analysis targeting class III SnRK2 protein kinase genes resulted in repression of the NAC gene, *RD26*, indicating that the expression of stress-inducible NACs is under control of the central ABA perception and signaling module (Nakashima et al., 2012; Fernando and Schroeder, 2016). Overexpression of the stress responsive, NAC gene, *SNAC1*, has been reported to lead to salt and drought tolerance in rice without a penalty in yield (Hu et al., 2006). SNAC1 was shown to bind to the promoter of the stress-induced gene, early responsive to drought 1 (*OsERD1*), and many stress-related genes were up-regulated in the *SNAC1*-overexpressing rice plants. The transgenic lines were also more sensitive to ABA and showed reduced water loss due to enhanced stomatal closing (with apparent no effect in photosynthesis), possibly by drought induction of *SNAC1* in guard cells (Hu et al., 2006). Also, OsNAC5 has been found to improve drought tolerance in rice via up-regulation of stress-inducible genes, and both OsNAC6 and OsNAC10 may also improve rice drought tolerance (reviewed by Nakashima et al., 2014; Singh and Laxmi, 2015).

Basic helix-loop-helix (bHLH) TFs have been implicated in drought regulation of stress-related genes via a wide-range of possible tolerance mechanisms, including stomatal development, ABA signaling, trichome and root hair development, osmoregulation, photosynthesis and growth regulation, in addition to ROS scavenging (reviewed by Castilhos et al., 2015; Sun et al., 2018). For example, AtMYC2 (bHLH) and AtMYB2 (MYB) TFs interact with *cis* elements in the promoter of the dehydration-responsive gene, *rd22*, to function as transcriptional activators in ABA-inducible gene expression under drought stress in Arabidopsis (Abe et al., 2003). However, strong alterations of stomatal development elicited by some bHLH TFs may hinder their practical application in cultivar development (Castilhos et al., 2015).

The bHLH family member, PTF1, has been found to play a role in low-Pi tolerance in rice, maize and soybean (see section “TFs Involved in Plant Low-P response/P Efficiency”). In maize, ZmPTF1 was also shown to enhance lateral root development and confer drought tolerance (Li Z. et al., 2019). Enhanced drought tolerance in the *ZmPTF1*-overexpression lines might be a result of activation of ABA and auxin signaling pathways and enhanced lateral root growth, which may be at least in part caused by up-regulation of NAC TFs. In fact, ZmPTF1 was shown to bind to the promoter of *NAC30* and other TFs, acting as a positive regulator of those genes (Li Z. et al., 2019). Thus, a possible connection between NAC-mediated tolerance to both drought and low-P conditions may be mediated at some extent via bHLH-dependent regulation of NAC TFs.

Interestingly, NAC TFs may also connect with Al tolerance based on up-regulation of *VuNAR1* in the *Vigna umbellata* root apex (Lou et al., 2020) and Al inducibility of other NACs (Escobar-Sepúlveda et al., 2017; Jin et al., 2020). Since VuNAR1 was shown to bind to the promoters of wall-associated receptor kinase genes, this NAC gene may confer Al tolerance through regulation of cell wall pectin metabolism (Lou et al., 2020). Interestingly, this mechanism could possibly feedback on P acquisition, since wall-associated kinases have been shown to affect root growth (Kanneganti and Gupta, 2008, 2011; Kaur et al., 2013).

### MYB TFs

Transcription factors possessing a conserved MYB domain involved with DNA binding are important players in abiotic stress tolerance and are intimately involved in cross-talk between different types of abiotic stresses. MYB TFs may influence drought tolerance via regulation of root growth and development, leaf development, stomatal movement in response to drought, cell wall biosynthesis, cuticle and suberin biosynthesis, and antioxidant activity via accumulation of flavonoids (reviewed by Baldoni et al., 2015). MYB TFs are also closely associated with changes in root morphology, which involves rather complex responses to different hormones. MYB77 has been implicated in auxin signaling via interaction with auxin response factors (ARFs), changing lateral root growth (Shin et al., 2007). MYB involvement with ABA signaling stems from the interaction between the ABA sensing gene, PLY8, and MYB77 (Zhao et al., 2014). By increasing auxin signaling, this interaction leads to a recovery of lateral root growth following inhibition by ABA. An important role for MYB TFs in abiotic stress cross-talk is also suggested by the joint role of AtMYB60 (Oh et al., 2011) and AtMYB96 in both stomatal movement and lateral root growth, with an integrative role in ABA and auxin signaling being proposed for AtMYB96 (Baldoni et al., 2015). Another MYB transcription factor working in a similar way is SiMYB75, which function in an ABA-dependent manner to promote root growth and drought tolerance, which results from enhanced stomatal closure to reduce water loss (Dossa et al., 2020).

By far the most compelling case of a MYB transcription factor jointly modulating drought stress and P deficiency tolerance arises from AtMYB2 regulation of *miR399* (Baek et al., 2016, 2017). As previously described (Section 3), *miR399* is a key component in P homeostasis (Fujii et al., 2005; Baek et al., 2013). Baek et al. (2016) have shown that AtMYB2 regulation of miR399 is involved in drought responses, with transgenic Arabidopsis plants overexpressing *miR399f* exhibiting ABA resistance and drought hypersensitivity. This response is thought to be a consequence of ABA-signaling, with *miR399* targeting CSP41B and ABF3 (Baek et al., 2016).

### WRKY TFs

WRKY TFs can act both as activators or repressors of gene expression and are involved both with abiotic and biotic stress responses (Bakshi and Oelmüller, 2014). The function of WRKY genes in drought tolerance is closely related to ABA signaling, which gives rise to a multi-pronged mode of action on plant performance under drought including stomatal closure and changes in RSA. Studies with a promoter::reporter gene construct for the sorghum member of the WRKY family, SbWRKY30, indicated that the *SbWRKY30* promoter responds to different phytohormones such as ABA, GA and Me-JA (Yang et al., 2020a). Expressed both in the tap root and leaf, *SbWKRY30* was induced by drought stress and conferred drought tolerance both in Arabidopsis and rice by affecting RSA. This drought tolerance response may also be a result of enhanced ROS scavenging elicited by SbWRKY30. This transcription factor was also found to influence the transcription of a number of stress-responsive genes, including *SbRD29* (Yang et al., 2020a).

Among other WRKY proteins, AtWRKY40 has been shown to interact with the C-terminal of the ABA receptor ABAR, with ABA acting to remobilize AtWRKY40 from the nucleus to the cytoplasm (reviewed by Rushton et al., 2012). Since AtWRKY40, AtWRKY18 and AtWRKY60 are negative regulators of ABA signaling, this mechanism leads to de-repression of ABA-dependent pathways and, hence, induction of ABA-responsive genes (Shang et al., 2010). With ABA sensing by its receptors, ABI5 is de-repressed and activates AtWRKY63, which activates stress-inducible genes such as *RD29* and *COR47* (Ren et al., 2010; Rushton et al., 2012).

Although there are many reports of WRKY TFs influencing drought responses (Rushton et al., 2012; Rabara et al., 2014; Singh and Laxmi, 2015), examples of common WRKY proteins also acting on Al tolerance and P deficiency tolerance are rather scarce, which might merely reflect less research focus on the involvement of WRKY TFs in abiotic stresses other than drought. Transgenic Arabidopsis with constitutive expression of the stress-response transcriptional coactivator, *multiprotein bridging factor 1c* (*MBF1c*), were more tolerant to bacterial infection, heat, and osmotic stress (Suzuki et al., 2005). *AtWRKY46* expression was found to be elevated in the transgenic lines, albeit only slightly, suggesting that AtWRKY46 could possibly be involved with stress tolerance in the *MBF1c* lines. Somewhat more compelling is the observation that *AtWRKY46* was induced by drought, salt and oxidative stress (Ding et al., 2014). AtWRKY46 was expressed in guard cells and may regulate stomatal opening and drought stress. Additionally, AtWRKY46 was found to regulate lateral root development in osmotic and salt stress conditions, possibly via ABA-signaling (in part via ABI4 regulation) and auxin homeostasis (Ding et al., 2015).

This suggests that AtWRKY46 could act on stress tolerance beyond its repressor role on the expression of the Al tolerance gene, *AtALMT1* (Ding et al., 2013), possibly influencing P acquisition and tolerance to drought stress. While enhanced lateral roots by AtWRKY46 might be expected to lead to enhanced P acquisition on low-P conditions, the nature of the impact of AtWRKY46 on drought tolerance, whether negative or positive, is yet to be unraveled in detail. Another possible case of cross-talk between drought stress tolerance and tolerance to low-P conditions involves WRKY45. Arabidopsis lines overexpressing *OsWRKY45* showed enhanced disease resistance and drought tolerance, possibly via ABA-mediated stomatal closure and induction of stress-related genes (Qiu and Yu, 2009). In Arabidopsis, *AtWRKY45* is induced by low-P, regulates *PSI* genes and is involved with changes in RSA that are apparently P-independent (see section “TFs Involved in Plant Low-P response/P Efficiency”; Devaiah et al., 2007). Also, AtWRKY45 participates in P responses by binding to the *PHT1;1* promoter and regulating its transcription, thereby enhancing P uptake (see section “TFs Involved in Plant Low-P response/P Efficiency”; Wang et al., 2014). These reports suggest that WRKY45 could have a pleiotropic effect, enhancing both drought tolerance and P acquisition in low-P conditions.

### Ethylene Response Factors (ERFs)

Ethylene response factors are TFs in the AP2/ERF superfamily, which are involved in tolerance to multiple abiotic stresses (Debbarma et al., 2019). Well-known members of this family include the Dehydration Responsive Element Binding (DREB) factors, which are frequently involved in the ABA-independent regulation of drought responsive genes (Singh and Laxmi, 2015), possibly involving ethylene signaling (Leng and Zhao, 2019). However, in some cases, DRE elements on some promoters are involved in both ABA-dependent and ABA-independent abiotic stresses (Agarwal et al., 2017). Overexpression of DREB TFs has resulted in increased drought tolerance, but with occasional deleterious side-effects (Agarwal et al., 2017). Chen Y. et al. (2018) identified in *Jatropha curcas* a P starvation responsive AP2/ERF transcription factor, *JcERF035*, which was downregulated under -P conditions. Overexpression of this P-starvation responsive AP2/ERF in Arabidopsis resulted in enhanced root hairs but reduced number and length of lateral roots, which was apparently independent from P supply. Recently, overexpression of *ERF74* in Arabidopsis was shown to enhance tolerance to a variety of stress factors, including drought, high light, heat and Al toxicity, whereas the *erf74* mutant displayed higher sensitivity to these stresses. Like many other abiotic stresses, Al toxicity, generates ROS (see Section 2.2a), which may both be a toxic product and also can be a signal. Yao et al. (2017) showed that the *erf74* mutant lacked the reactive oxygen species burst often seen in the early stages of various stresses, which was due to lower expression level *of RESPIRATORY BURST OXIDASE HOMOLOG D (RbohD*) in the *erf74* mutant. Possibly this is part of a ROS signaling pathway conditioning tolerance to stresses such as Al toxicity, drought and temperature extremes, which may be related to ROS signals (Yao et al., 2017). While these studies with JcERF35 and AtERF74 suggest that some ERF transfactors might be involved in plant responses to P, Al and drought stress, this area awaits considerable further investigation. Also, given the involvement of ABA as a positive regulator of Al tolerance, including induction of *AtALMT1* and *AtMATE* expression (see Section 2.2b), other, yet unidentified factors may connect drought and Al tolerance via ABA-dependent pathways.

## Conclusion

**FIGURE 1:**
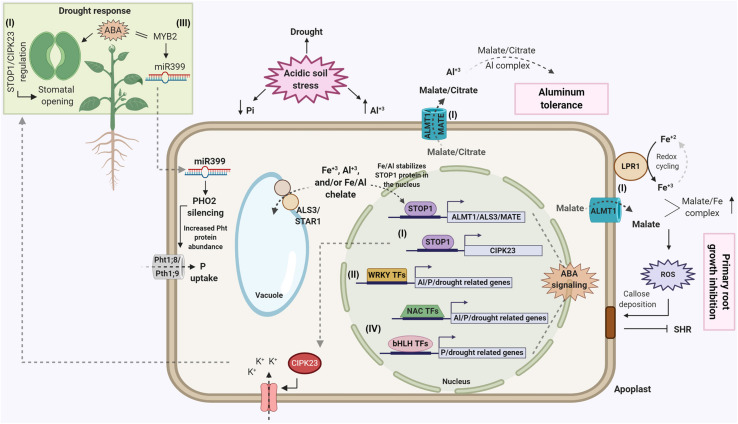
Model for cross-talk between Al toxicity, low-P availability and drought stress. Four transcriptional regulatory networks are highlighted that we identified in this review that may be involved with plant responses to these three abiotic stresses. **(I)** The *SENSITIVE TO PROTON RHIZOTOXICITY 1* (*STOP1*) transcription factor is involved in Al tolerance, P deficiency and drought stress. In addition to *AtMATE*, *STOP1* transcriptionally activates the *ALUMINUM ACTIVATED MALATE TRANSPORTER1* (*ALMT1)* which encodes the root PM anion channel that mediates Al activated malate release from roots, detoxifying Al in the rhizosphere. P deficiency induces the release of the multicopper ferroxidase (LPR1) that reduces/oxidizes Fe. P deficiency also induces expression of *ALMT1*, and the malate released via the ALMT1 protein chelates and facilitates accumulation of Fe in the cell wall where its oxidoreduction is catalyzed by LPR1, generating peroxides, which triggers lignification and cell wall stiffening, and rapid inhibition of root growth. At the same time, ROS generated from oxidoreduction of Fe triggers callose formation, which blocks plasmodesmata between stem cell initials and cells of the RAM outside the stem cell niche. This blockage of the plasmodesmata prevents cell-to-cell transport of the transcription factor, *SHORT-ROOT*, which is needed to drive stem cell division. This exhausts the RAM and terminates primary root growth. The Fe accumulated in the cell wall also drives increased Fe influx into the cytoplasm of RAM and the increased Fe helps stabilize and enhance STOP1 accumulation in the nucleus. Under P sufficient conditions, the tonoplast ABC transporter, ALS3/STAR1, is hypothesized to transport the Fe or Al (depending on soil conditions) or possibly Fe/Al-chelates into the vacuole and the reduction of Fe/Al levels in the cytoplasm and nucleus represses STOP1 protein accumulation in the nucleus, inhibiting *ALMT1*, transcriptional activation. With regards to STOP1’s involvement in drought response, *STOP1* also transcriptionally activates *CBL-INTERACTING PROTEIN KINASE 23* (*CIPK23*) expression, and the CIPK23 protein is an activator of high affinity K^+^ transporters in the root and possibly the shoot, which could result in enhanced stomatal opening and a reduction in drought tolerance due to increased transpirational water loss. **(II)** A second family of candidate TFs are the large WRKY family. There are several WRKY transcription factor family members involved in responses to drought and Al toxicity. For example, *AtWRKY46* represses *ALMT1* expression in the absence of Al and also is expressed in guard cells in response to drought stress; although its role in drought response remains to be elucidated **(III)** The AtMYB2 transcription factor co-regulates P efficiency and is involved in drought response via regulation of *miR399*. AtMYB2 induces *miR399* in response to ABA and salt stress, and overexpression of *miR399* results in salt and ABA tolerance but interestingly, is associated with drought sensitivity. *miR399* plays a well-documented role in long distance P deficiency signaling in the phloem as it is synthesized in response to P deficiency in mature source leaves and is translocated in the phloem to the root, where it silences *PHOSPHATE 2* (*PHO2)*, which encodes a ubiquitin-conjugating E2 enzyme that negatively regulates P transporters under P sufficiency. **(IV)** NAC and Basic helix-loop-helix (bHLH) transcriptional factors have been implicated in drought regulation of stress-related genes via a wide-range of possible tolerance mechanisms, which involves ABA-dependent and independent pathways. The bHLH family member, PTF1, plays a role in low P tolerance, enhancing lateral root development. ZmPTF1 bind to the promoter of NAC30 and other TFs, acting as a positive regulator of those genes. Thus, a possible connection between NAC-mediated tolerance to both drought and low-P conditions may be mediated at some extent via bHLH-dependent regulation of NAC TFs. NAC TFs may also connect with Al tolerance based on up-regulation of VuNAR1 root apex and Al inducibility of other NACs. VuNAR1 also binds to the promoters of wall-associated receptor kinase genes, conferring Al tolerance through regulation of cell wall pectin metabolism. Although there is no evidence for direct interaction of STOP1, *AtMATE1* and *AtALS3* promoters, it is clear that STOP1 is crucial for induction of these genes. Not shown here is a possible link between Al tolerance and drought tolerance via ERF transcription factors, whose supporting evidence is more limited and preliminary. The figure was created with BioRender.com.

In this review, we examined the literature for common elements shared between the three major stresses that often co-occur on acidic soils: Al toxicity, low P availability and drought, with a focus on genes/proteins involved in signaling and/or regulatory pathways and networks controlling plant responses and adaptations to these three abiotic stresses. In general, research on crop adaptation to acidic soils has focused on one of the two primary stresses resulting from the unique chemistry of acidic soils, Al toxicity or P deficiency. But as this field has advanced and matured, we showed here that quite recently, a number of genes have been identified that are involved both in Al resistance and adaptation to P deficiency. The very broad field of research on crop adaptations to drought, on the other hand, has not focused much on the possible role of drought resistance mechanisms in adaptations to acidic soils. This is primarily because drought, by its very nature, occurs on all types of soils in many different eco-agricultural systems. Nonetheless, in this review we did identify several genes and the proteins they encode that could play a role in adaptation to all three of these abiotic stresses. At the beginning of this review, we speculated that genes involved in signaling/regulatory networks might be the best source for candidates involved in crop adaptation to all three stresses. This turned out to be the case as we identified transcription factors from several TF families that could play this pleiotropic role. These TF’s are summarized here and a model of the function of the best candidate TF’s in the three abiotic stresses is presented in Figure 1.

(1) STOP1, a C2H2 Zinc Finger Transcription Factor: This is probably the most interesting candidate as it has been shown to clearly be a key Al tolerance gene, regulating the Al-induced transcriptional activation of a number of Al tolerance genes in Arabidopsis, including the major Al tolerance gene, *ALMT1* (Iuchi et al., 2007). STOP1 also plays a key role in Arabidopsis root response to P deficiency, as under low P STOP1 again activates *ALMT1* expression, enhancing the production of the *ALMT1* anion channel that facilitates root malate release, which is central to the root apex Fe accumulation needed to exhaust the primary root RAM and root growth (Müller et al., 2015; Balzergue et al., 2017; Mora-Macías et al., 2017). The loss of primary root apical dominance then appears to lead to enhanced lateral root growth which could confer enhanced P acquisition in low P soil. Additionally, there is a reasonable amount of circumstantial evidence implicating STOP1 in several drought responses. This includes the recent finding that *stop1* knockout lines showed enhanced drought tolerance in Arabidopsis (Sadhukhan et al., 2019), suggesting that STOP1 is a negative regulator of drought tolerance. These authors also found that the STOP1 regulated the expression of the gene encoding the CBL-interacting protein kinase 23 (CIPK23), and complementation of the *stop1* mutant with CIPK23 reversed the drought phenotype (back to drought sensitivity). Furthermore, in a heterologous system, *Xenopus* oocytes, CIPK23 can activate via phosphorylation the guard cell PM anion channel, SLAC1 (SLOW ANION CHANNEL-ASSOCIATED 1) (Maierhofer et al., 2014), the direct link between this process and drought physiology involving stomatal closure is still unclear. Nevertheless, these findings all point toward the highly pleiotropic nature of the transcription factor, *STOP1*.
(2) WRKY Transcription factors: We found different members of the WRKY TF family that were involved in all three stresses, but did not identify a single WRKY member clearly influencing all stresses. One possibility is AtWRKY46, which is involved in Al tolerance where it represses *ALMT1* expression in the absence of Al (Ding et al., 2013). Additionally, it was shown to be involved in drought response, as it is expressed in guard cells and this expression is induced by drought, salt and oxidative stress (Ding et al., 2014). Additionally, AtWRKY46 was found to enhance lateral root development in response to osmotic and salt stress conditions, possibly via ABA-signaling and auxin homeostasis (Ding et al., 2015). It would be interesting to find out if this increased lateral root development and growth could also play a role in improved P efficiency.
(3) AtMYB2 Regulation of *miR399*: There is good evidence for this MYB transcription factor co-regulating P efficiency and drought tolerance via regulation of *miR399* (Baek et al., 2016, 2017). As detailed above, *miR399* is an important player in P homeostasis via a long distance systemic signaling system in the phloem translocating *miR399* from mature source leaves to the root, where it silences *PHO2*, the ubiquitin-conjugating E2 enzyme that negatively regulates P transporters under P sufficiency (Fujii et al., 2005; Baek et al., 2013). It was also shown that AtMYB2 regulation of *miR399* is also involved in plant responses to abscisic acid (ABA), and to salt and drought (Baek et al., 2016). Salt and ABA treatment induced the expression of *miR399*, and overexpression of *miR399* resulted in enhanced salt tolerance and interestingly, hypersensitivity to drought. Hence the pleiotropic nature of AtMYB regulation of *miR399* spanning low P and drought stress is apparent, although the functional basis of its impact on drought responses remains to be elucidated.

In conclusion, there are tantalizing links in the literature between the regulation of plant adaptation and responses to Al toxicity, P deficiency and drought stress. To provide the readers with a summary of the work reviewed in this paper, we have included Supplementary Table 1 (Gene families possibly involved in pleiotropic mechanisms) resulting in multiple stress tolerances (tolerance to Al toxicity, P deficiency and drought) which contains lists of members of five gene families (*WRKY*, *STOP1*, *MYB*, *bHLH*, and *NAC*) involved in Al toxicity, drought stress and P deficiency. However more research is needed to say with certainty that the same factors can regulate tolerance to all three stresses. If that is the case, it will be quite intriguing to determine if these genes would be useful in a plant breeding program for multiple environmental stresses. Within a scenario where the same genes in some way influence tolerance to multiple stresses, instances of conflicting effects may be foreseen, as previously discussed. If the same gene acts simultaneously as positive and negative regulators of tolerance to different stresses, this may cancel its final effect in phenotypic expression or even be detrimental to crop performance on acidic soils. Within the context of molecular breeding strategies targeting quantitative traits such as genomic selection, these and other negative effects may be filtered out along the selection process via genomic estimation of breeding values. However, transgenic approaches may be more sensitive to this problem. In that regard, gene editing has emerged as a powerful approach to fine tune gene expression, which could help circumvent negative effects coming from pleiotropy or epistasis. This approach has proven useful in bypassing negative epistasis effects on yield in tomato (Soyk et al., 2017) and to tackle quantitative trait variation (Rodríguez-Leal et al., 2017). Particularly to deal with possible hurdles when exploring pleiotropy in crop adaption to acidic soils, it is more than certain that in-depth knowledge of the physiological and genetic underpinnings of multiple stress tolerance will be required.

## Author Contributions

JM and LK also handled the revising and editing of the full version of the manuscript. All authors contributed by conducting literature research and writing of different parts of the text.

## Conflict of Interest

The authors declare that the research was conducted in the absence of any commercial or financial relationships that could be construed as a potential conflict of interest.

## References

[B1] Abe H. Urao T. Ito T. Seki M. Shinozaki K. (2003). Arabidopsis AtMYC2 (bHLH) and AtMYB2 (MYB) function as transcriptional activators in abscisic acid signaling. *Society* 15 63–78. 10.1105/tpc.006130.salt PMC143451 12509522

[B2] Agarwal P. K. Gupta K. Lopato S. Agarwal P. (2017). Dehydration responsive element binding transcription factors and their applications for the engineering of stress tolerance. *J. Exp. Bot.* 68 2135–2148. 10.1093/jxb/erx118 28419345

[B3] Aravind J. Rinku S. Pooja B. Shikha M. Kaliyugam S. Mallikarjuna M. G. (2017). Identification, characterization, and functional validation of drought-responsive microRNAs in subtropical maize inbreds. *Front. Plant Sci.* 8:941. 10.3389/fpls.2017.00941 28626466 PMC5454542

[B4] Arenhart R. A. Bai Y. Valter De Oliveira L. F. Bucker Neto L. Schunemann M. Maraschin F. D. S. (2014). New insights into aluminum tolerance in rice: the ASR5 protein binds the STAR1 promoter and other aluminum-responsive genes. *Mol. Plant* 7 709–721. 10.1093/mp/sst160 24253199 PMC3973494

[B5] Arenhart R. A. De Lima J. C. Pedron M. Carvalho F. E. L. Da Silveira J. A. G. Rosa S. B. (2013). Involvement of ASR genes in aluminium tolerance mechanisms in rice. *Plant Cell Environ.* 36 52–67. 10.1111/j.1365-3040.2012.02553.x 22676236

[B6] Arenhart R. A. Schunemann M. Bucker Neto L. Margis R. Wang Z. Y. Margis-Pinheiro M. (2016). Rice ASR1 and ASR5 are complementary transcription factors regulating aluminium responsive genes. *Plant Cell Environ.* 39 645–651. 10.1111/pce.12655 26476017 PMC7256019

[B7] Aukerman M. J. Sakai H. (2003). Regulation of flowering time and floral organ identity by a microRNA and its APETALA2-like target genes. *Plant Cell* 15 2730–2741. 10.1105/tpc.016238 14555699 PMC280575

[B8] Aung K. Lin S.-I. Wu C.-C. Huang Y.-T. Su C. Chiou T.-J. (2006). pho2, a phosphate overaccumulator, is caused by a nonsense mutation in a microRNA399 target gene. *Plant Physiol.* 141 1000–1011. 10.1104/pp.106.078063 16679417 PMC1489903

[B9] Azevedo G. C. Cheavegatti-Gianotto A. Negri B. F. Hufnagel B. da Silva L. C. E. Magalhaes J. V. (2015). Multiple interval QTL mapping and searching for PSTOL1 homologs associated with root morphology, biomass accumulation and phosphorus content in maize seedlings under low-P. *BMC Plant Biol.* 15:172. 10.1186/s12870-015-0561-y 26148492 PMC4492167

[B10] Baek D. Chun H. J. Kang S. Shin G. Park S. J. Hong H. (2016). A role for arabidopsis miR399f in salt, drought, and ABA signaling. *Mol. Cells* 39 111–118. 10.14348/molcells.2016.2188 26674968 PMC4757798

[B11] Baek D. Chun H. J. Yun D. J. Kim M. C. (2017). Cross-talk between phosphate starvation and other environmental stress signaling pathways in plants. *Mol. Cells* 40 697–705. 10.14348/molcells.2017.0192 29047263 PMC5682247

[B12] Baek D. Kim M. C. Chun H. J. Kang S. Park H. C. Shin G. (2013). Regulation of miR399f transcription by AtMYB2 affects phosphate starvation responses in *Arabidopsis*. *Plant Physiol.* 161 362–373. 10.1104/pp.112.205922 23154535 PMC3532267

[B13] Baillo E. H. Kimotho R. N. Zhang Z. Xu P. (2019). Transcription factors associated with abiotic and biotic stress tolerance and their potential for crops improvement. *Genes (Basel)* 10 1–23. 10.3390/genes10100771 31575043 PMC6827364

[B14] Bakhshi B. Fard E. M. Nikpay N. Ebrahimi M. A. Bihamta M. R. Mardi M. (2016). MicroRNA signatures of drought signaling in rice root. *PLoS One* 11:e0156814. 10.1371/journal.pone.0156814 27276090 PMC4898717

[B15] Bakshi M. Oelmüller R. (2014). Wrky transcription factors jack of many trades in plants. *Plant Signal. Behav.* 9:e27700. 10.4161/psb.27700 24492469 PMC4091213

[B16] Baldoni E. Genga A. Cominelli E. (2015). Plant MYB transcription factors: Their role in drought response mechanisms. *Int. J. Mol. Sci.* 16 15811–15851. 10.3390/ijms160715811 26184177 PMC4519927

[B17] Balzergue C. Dartevelle T. Godon C. Laugier E. Meisrimler C. Teulon J. M. (2017). Low phosphate activates STOP1-ALMT1 to rapidly inhibit root cell elongation. *Nat. Commun.* 8 1–16. 10.1038/ncomms15300 28504266 PMC5440667

[B18] Bano A. Dorffling K. Bettin D. Hahn H. (1993). Abscisic acid and cytokinins as possible root-to-shoot signals in xylem sap of rice plants in drying soil. *Funct. Plant Biol.* 20 109–115. 10.1071/pp9930109

[B19] Banti V. Mafessoni F. Loreti E. Alpi A. Perata P. (2010). The heat-inducible transcription factor HsfA2 enhances anoxia tolerance in *Arabidopsis*. *Plant Physiol.* 152 1471–1483. 10.1104/pp.109.149815 20089772 PMC2832282

[B20] Barberon M. Vermeer J. E. M. De Bellis D. Wang P. Naseer S. Andersen T. G. (2016). Adaptation of root function by nutrient-induced plasticity of endodermal differentiation. *Cell* 164 447–459. 10.1016/j.cell.2015.12.021 26777403

[B21] Bari R. Pant B. D. Stitt M. Scheible W. R. (2006). PHO2, microRNA399, and PHR1 define a phosphate-signaling pathway in plants. *Plant Physiol.* 141 988–999. 10.1104/pp.106.079707 16679424 PMC1489890

[B22] Bariola P. A. Howard C. J. Taylor C. B. Verburg M. T. Jaglan V. D. Green P. J. (1994). The *Arabidopsis* ribonuclease gene RNS1 is tightly controlled in response to phosphate limitation. *Plant J.* 6 673–685. 10.1046/j.1365-313X.1994.6050673.x 8000425

[B23] Basu U. Good A. G. Taylor G. J. (2001). Transgenic *Brassica napus* plants overexpressing aluminium-induced mitochondrial manganese superoxide dismutase cDNA are resistant to aluminium. *Plant. Cell Environ.* 24 1269–1278.

[B24] Batool A. Cheng Z.-G. Akram N. A. Lv G.-C. Xiong J.-L. Zhu Y. (2019). Partial and full root-zone drought stresses account for differentiate root-sourced signal and yield formation in primitive wheat. *Plant Methods* 15:75. 10.1186/s13007-019-0461-5 PMC6624928 31338115

[B25] Belda-Palazon B. Gonzalez-Garcia M.-P. Lozano-Juste J. Coego A. Antoni R. Julian J. (2018). PYL8 mediates ABA perception in the root through non-cell-autonomous and ligand-stabilization–based mechanisms. *Proc. Natl. Acad. Sci. U.S.A.* 115 E11857—E11863. 10.1073/pnas.1815410115 PMC6294950 30482863

[B26] Bernardino K. C. Pastina M. M. Menezes C. B. De Sousa S. M. Maciel L. S. Geraldo Carvalho G. C. (2019). The genetic architecture of phosphorus efficiency in sorghum involves pleiotropic QTL for root morphology and grain yield under low phosphorus availability in the soil. *BMC Plant Biol.* 19:87. 10.1186/s12870-019-1689-y 30819116 PMC6394046

[B27] Bhattacharjee S. (2012). The language of reactive oxygen species signaling in plants. *J. Bot.* 2012 985298.

[B28] Blackman P. G. Davies W. J. (1985). Root to shoot communication in maize plants of the effects of soil drying. *J. Exp. Bot.* 36 39–48. 10.1093/jxb/36.1.39

[B29] Blokhina O. Virolainen E. Fagerstedt K. V. (2003). Antioxidants, oxidative damage and oxygen deprivation stress: a review. *Ann. Bot.* 91 179–194. 10.1093/aob/mcf118 12509339 PMC4244988

[B30] Blum A. (2017). Osmotic adjustment is a prime drought stress adaptive engine in support of plant production. *Plant Cell Environ.* 40 4–10. 10.1111/pce.12800 27417527

[B31] Castilhos G. Lazzarotto F. Spagnolo-Fonini L. Bodanese-Zanettini M. H. Margis-Pinheiro M. (2015). Possible roles of basic helix-loop-helix transcription factors in adaptation to drought. *Plant Sci.* 235:130 10.1016/j.plantsci.2015.03.012 24767109

[B32] Chen D. Chai S. McIntyre C. L. Xue G.-P. (2018). Overexpression of a predominantly root-expressed NAC transcription factor in wheat roots enhances root length, biomass and drought tolerance. *Plant Cell Rep.* 37 225–237. 10.1007/s00299-017-2224-y 29079898

[B33] Chen J. Wang Y. Wang F. Yang J. Gao M. Li C. (2015). The rice CK2 kinase regulates trafficking of phosphate transporters in response to phosphate levels. *Plant Cell* 27 711–723. 10.1105/tpc.114.135335 25724641 PMC4558666

[B34] Chen Y. Wu P. Zhao Q. Tang Y. Chen Y. Li M. (2018). Overexpression of a phosphate starvation response ap2/erf gene from physic nut in arabidopsis alters root morphological traits and phosphate starvation-induced anthocyanin accumulation. *Front. Plant Sci.* 9:1186. 10.3389/fpls.2018.01186 30177937 PMC6109760

[B35] Chen Y. F. Li L. Q. Xu Q. Kong Y. H. Wang H. Wu W. H. (2009). The WRKY6 transcription factor modulates PHOSPHATE1 expression in response to low pi stress in arabidopsis. *Plant Cell* 21 3554–3566. 10.1105/tpc.108.064980 19934380 PMC2798333

[B36] Chen Z. C. Yamaji N. Motoyama R. Nagamura Y. Ma J. F. (2012). Up-regulation of a magnesium transporter gene osmgt1 is required for conferring aluminum tolerance in rice. *Plant Physiol.* 159 1624–1633. 10.1104/pp.112.199778 22732245 PMC3425201

[B37] Chen Z. C. Yokosho K. Kashino M. Zhao F. J. Yamaji N. Ma J. F. (2013). Adaptation to acidic soil is achieved by increased numbers of cis-acting elements regulating ALMT1 expression in *Holcus lanatus*. *Plant J.* 76 10–23. 10.1111/tpj.12266 23773148

[B38] Chien P. S. Chiang C. P. Leong S. J. Chiou T. J. (2018). Sensing and signaling of phosphate starvation: from local to long distance. *Plant Cell Physiol.* 59 1714–1722. 10.1093/pcp/pcy148 30053262

[B39] Chiou T.-J. Aung K. Lin S.-I. Wu C.-C. Chiang S.-F. Su C. (2006). Regulation of phosphate homeostasis by microRNA in *Arabidopsis*. *Plant Cell* 18 412–421. 10.1105/tpc.105.038943 16387831 PMC1356548

[B40] Christmann A. Weiler E. W. Steudle E. Grill E. (2007). A hydraulic signal in root-to-shoot signalling of water shortage. *Plant J.* 52 167–174. 10.1111/j.1365-313x.2007.03234.x 17711416

[B41] Cutler S. R. Rodriguez P. L. Finkelstein R. R. Abrams S. R. (2010). Abscisic acid: emergence of a core signaling network. *Annu. Rev. Plant Biol.* 61 651–679. 10.1146/annurev-arplant-042809-112122 20192755

[B42] Dai X. Wang Y. Yang A. Zhang W. H. (2012). OsMYB2P-1, an R2R3 MYB transcription factor, is involved in the regulation of phosphate-starvation responses and root architecture in rice. *Plant Physiol.* 159 169–183. 10.1104/pp.112.194217 22395576 PMC3375959

[B43] Dai X. Wang Y. Zhang W. H. (2016). OsWRKY74, a WRKY transcription factor, modulates tolerance to phosphate starvation in rice. *J. Exp. Bot.* 67 947–960. 10.1093/jxb/erv515 26663563 PMC4737085

[B44] Daspute A. A. Kobayashi Y. Panda S. K. Fakrudin B. Kobayashi Y. Tokizawa M. (2018). Characterization of CcSTOP1; a C2H2-type transcription factor regulates Al tolerance gene in pigeonpea. *Planta* 247 201–214. 10.1007/s00425-017-2777-6 28921050

[B45] De Sousa S. M. Clark R. T. Mendes F. F. Carlos De Oliveira A. Vilaça De Vasconcelos M. J. Parentoni S. N. (2012). A role for root morphology and related candidate genes in P acquisition efficiency in maize. *Funct. Plant Biol.* 39 925–935. 10.1071/FP12022 32480842

[B46] Debbarma J. Sarki Y. N. Saikia B. Boruah H. P. D. Singha D. L. Chikkaputtaiah C. (2019). Ethylene Response Factor (ERF) family proteins in abiotic stresses and CRISPR–Cas9 genome editing of ERFs for multiple abiotic stress tolerance in crop plants: a review. *Mol. Biotechnol.* 61 153–172. 10.1007/s12033-018-0144-x 30600447

[B47] Devaiah B. N. Karthikeyan A. S. Raghothama K. G. (2007). WRKY75 transcription factor is a modulator of phosphate acquisition and root development in *Arabidopsis*. *Plant Physiol.* 143 1789–1801. 10.1104/pp.106.093971 17322336 PMC1851818

[B48] Dietrich D. Pang L. Kobayashi A. Fozard J. A. Boudolf V. Bhosale R. (2017). Root hydrotropism is controlled via a cortex-specific growth mechanism. *Nat. Plants* 3:17057. 10.1038/nplants.2017.57 28481327

[B49] Ding Z. J. Yan J. Y. Li C. X. Li G. X. Wu Y. R. Zheng S. J. (2015). Transcription factor WRKY46 modulates the development of *Arabidopsis* lateral roots in osmotic/salt stress conditions via regulation of ABA signaling and auxin homeostasis. *Plant J.* 84 56–69. 10.1111/tpj.12958 26252246

[B50] Ding Z. J. Yan J. Y. Xu X. Y. Li G. X. Zheng S. J. (2013). WRKY46 functions as a transcriptional repressor of ALMT1, regulating aluminum-induced malate secretion in *Arabidopsis*. *Plant J.* 76 825–835. 10.1111/tpj.12337 24118304

[B51] Ding Z. J. Yan J. Y. Xu X. Y. Yu D. Q. Li G. X. Zhang S. Q. (2014). Transcription factor WRKY46 regulates osmotic stress responses and stomatal movement independently in *Arabidopsis*. *Plant J.* 79 13–27. 10.1111/tpj.12538 24773321

[B52] Dodd I. C. Theobald J. C. Richer S. K. Davies W. J. (2009). Partial phenotypic reversion of ABA-deficient flacca tomato (*Solanum lycopersicum*) scions by a wild-type rootstock: normalizing shoot ethylene relations promotes leaf area but does not diminish whole plant transpiration rate. *J. Exp. Bot.* 60 4029–4039. 10.1093/jxb/erp236 19648172 PMC2755025

[B53] Dong J. Piñeros M. A. Li X. Yang H. Liu Y. Murphy A. S. (2017). An *Arabidopsis* ABC transporter mediates phosphate deficiency-induced remodeling of root architecture by modulating iron homeostasis in roots. *Mol. Plant* 10 244–259. 10.1016/j.molp.2016.11.001 27847325

[B54] Dossa K. Mmadi M. A. Zhou R. Liu A. Yang Y. Diouf D. (2020). Ectopic expression of the sesame MYB transcription factor SiMYB305 promotes root growth and modulates ABA-mediated tolerance to drought and salt stresses in *Arabidopsis*. *AoB Plants* 12 1–14. 10.1093/aobpla/plz081 PMC7019004 32099638

[B55] Driedonks N. Xu J. Peters J. L. Park S. Rieu I. (2015). Multi-level interactions between heat shock factors, heat shock proteins, and the redox system regulate acclimation to heat. *Front. Plant Sci.* 6:999. 10.3389/fpls.2015.00999 26635827 PMC4647109

[B56] Du Q. Wang K. Zou C. Xu C. Li W.-X. (2018). The PILNCR1-miR399 regulatory module is important for low phosphate tolerance in maize. *Plant Physiol.* 177 1743–1753. 10.1104/pp.18.00034 29967097 PMC6084674

[B57] Enomoto T. Tokizawa M. Ito H. Iuchi S. Kobayashi M. Yamamoto Y. Y. (2019). STOP1 regulates the expression of HsfA2 and GDH s that are critical for low-oxygen tolerance in *Arabidopsis*. *J. Exp. Bot.* 70 3297–3311. 10.1093/jxb/erz124 30882866

[B58] Escobar-Sepúlveda H. F. Trejo-Téllez L. I García-Morales S. Gómez-Merino F. C. (2017). Expression patterns and promoter analyses of aluminum-responsive NAC genes suggest a possible growth regulation of rice mediated by aluminum, hormones and NAC transcription factors. *PLoS One* 12:e0186084. 10.1371/journal.pone.0186084 29023561 PMC5638308

[B59] Ezaki B. Gardner R. C. Ezaki Y. Matsumoto H. (2000). Expression of aluminum-induced genes in transgenic *Arabidopsis* plants can ameliorate aluminum stress and/or oxidative stress. *Plant Physiol.* 122 657–666. 10.1104/pp.122.3.657 10712528 PMC58900

[B60] Fambrini M. Vernieri P. Toncelli M. L. Rossi V. D. Pugliesi C. (1995). Characterization of a wilty sunflower (*Helianthus annuus* L.) mutant: III. Phenotypic interaction in reciprocal grafts from wilty mutant and wild-type plants. *J. Exp. Bot.* 46 525–530. 10.1093/jxb/46.5.525 12432039

[B61] Fan W. Lou H. Q. Gong Y. L. Liu M. Y. Cao M. J. Liu Y. (2015). Characterization of an inducible C2H2-type zinc finger transcription factor Vu STOP 1 in rice bean (*Vigna umbellata*) reveals differential regulation between low pH and aluminum tolerance mechanisms. *New Phytol.* 208 456–468. 10.1111/nph.13456 25970766

[B62] Feng W. Lindner H. Robbins N. E. Dinneny J. R. (2016). Growing out of stress: the role of cell-and organ-scale growth control in plant water-stress responses. *Plant Cell* 28 1769–1782. 10.1105/tpc.16.00182 27503468 PMC5006702

[B63] Fernando V. C. D. Schroeder D. F. (2016). “Role of ABA in *Arabidopsis* salt, drought, and desiccation tolerance,” in *Abiotic and Biotic Stress in Plants-Recent Advances and Future Perspectives*, eds Shanker A. Shanker C. (London: IntechOpen).

[B64] Fuganti-Pagliarini R. Ferreira L. C. Rodrigues F. A. Molinari H. B. C. Marin S. R. R. Molinari M. D. C. (2017). Characterization of soybean genetically modified for drought tolerance in field conditions. *Front. Plant Sci.* 8:448. 10.3389/fpls.2017.00448 28443101 PMC5387084

[B65] Fujii H. Chinnusamy V. Rodrigues A. Rubio S. Antoni R. Park S.-Y. (2009). In vitro reconstitution of an abscisic acid signalling pathway. *Nature* 462 660–664. 10.1038/nature08599 19924127 PMC2803041

[B66] Fujii H. Chiou T. J. Lin S. I Aung K. Zhu J. K. (2005). A miRNA involved in phosphate-starvation response in *Arabidopsis*. *Curr. Biol.* 15 2038–2043. 10.1016/j.cub.2005.10.016 16303564

[B67] Gamuyao R. Chin J. H. Pariasca-Tanaka J. Pesaresi P. Catausan S. Dalid C. (2012). The protein kinase Pstol1 from traditional rice confers tolerance of phosphorus deficiency. *Nature* 488 535–539. 10.1038/nature11346 22914168

[B68] Gareau J. R. Lima C. D. (2010). The SUMO pathway: emerging mechanisms that shape specificity, conjugation and recognition. *Nat. Rev. Mol. Cell Biol.* 11 861–871. 10.1038/nrm3011 21102611 PMC3079294

[B69] Godfray H. C. J. Beddington J. R. Crute I. R. Haddad L. Lawrence D. Muir J. F. (2010). Food security: the challenge of feeding 9 billion people. *Science* 327 812–818. 10.1126/science.1185383 20110467

[B70] Godon C. Mercier C. Wang X. David P. Richaud P. Nussaume L. (2019). Under phosphate starvation conditions, Fe and Al trigger accumulation of the transcription factor STOP1 in the nucleus of *Arabidopsis* root cells. *Plant J.* 99 937–949. 10.1111/tpj.14374 31034704 PMC6852189

[B71] Gollan T. Schurr U. Schulze E.-D. (1992). Stomatal response to drying soil in relation to changes in the xylem sap composition of *Helianthus annuus*. I. The concentration of cations, anions, amino acids in, and pH of, the xylem sap. *Plant. Cell Environ.* 15 551–559. 10.1111/j.1365-3040.1992.tb01488.x

[B72] González E. Solano R. Rubio V. Leyva A. Paz-Ares J. (2005). PHOSPHATE TRANSPORTER TRAFFIC FACILITATOR1 is a plant-specific SEC12-related protein that enables the endoplasmic reticulum exit of a high-affinity phosphate transporter in *Arabidopsis*. *Plant Cell* 17 3500–3512. 10.1105/tpc.105.036640 16284308 PMC1315384

[B73] Gonzalez-Guzman M. Pizzio G. A. Antoni R. Vera-Sirera F. Merilo E. Bassel G. W. (2012). *Arabidopsis* PYR/PYL/RCAR receptors play a major role in quantitative regulation of stomatal aperture and transcriptional response to abscisic acid. *Plant Cell* 24 2483–2496. 10.1105/tpc.112.098574 22739828 PMC3406898

[B74] Grieneisen V. A. Xu J. Marée A. F. M. Hogeweg P. Scheres B. (2007). Auxin transport is sufficient to generate a maximum and gradient guiding root growth. *Nature* 449 1008–1013. 10.1038/nature06215 17960234

[B75] Hamburger D. Rezzonico E. Petétot J. M.-C. Somerville C. Poirier Y. (2002). Identification and characterization of the *Arabidopsis* PHO1 gene involved in phosphate loading to the xylem. *Plant Cell* 14 889–902. 10.1105/tpc.000745 11971143 PMC150690

[B76] Han G. Lu C. Guo J. Qiao Z. Sui N. Qiu N. (2020). C2H2 Zinc finger proteins: master regulators of abiotic stress responses in plants. *Front. Plant Sci.* 11:115. 10.3389/fpls.2020.00115 32153617 PMC7044346

[B77] Hartung W. Wilkinson S. Davies W. J. (1998). Factors that regulate abscisic acid concentrations at the primary site of action at the guard cell. *J. Exp. Bot.* 49 361–367. 10.1093/jexbot/49.suppl_1.361

[B78] Hauser F. Li Z. Waadt R. Schroeder J. I. (2017). SnapShot: abscisic acid signaling. *Cell* 171:1708. 10.1016/j.cell.2017.11.045 29245015 PMC5895850

[B79] He G.-H. Xu J.-Y. Wang Y.-X. Liu J.-M. Li P.-S. Chen M. (2016). Drought-responsive WRKY transcription factor genes TaWRKY1 and TaWRKY33 from wheat confer drought and/or heat resistance in *Arabidopsis*. *BMC Plant Biol.* 16:116. 10.1186/s12870-016-0806-4 27215938 PMC4877946

[B80] He H. Oo T. L. Huang W. He L.-F. Gu M. (2019). Nitric oxide acts as an antioxidant and inhibits programmed cell death induced by aluminum in the root tips of peanut (*Arachis hypogaea* L.). *Sci. Rep.* 9 1–12. 31267033 10.1038/s41598-019-46036-8 PMC6606607

[B81] He H.-Y. He L.-F. Gu M.-H. Li X.-F. (2012). Nitric oxide improves aluminum tolerance by regulating hormonal equilibrium in the root apices of rye and wheat. *Plant Sci.* 183 123–130. 10.1016/j.plantsci.2011.07.012 22195585

[B82] Henson I. E. Jensen C. R. Turner N. C. (1989). Leaf gas exchange and water relations of lupins and wheat. III. Abscisic acid and drought-induced stomatal closure. *Funct. Plant Biol.* 16 429–442. 10.1071/pp9890429

[B83] Hetz W. Hochholdinger F. Schwall M. Feix G. (1996). Isolation and characterization of rtcs, a maize mutant deficient in the formation of nodal roots. *Plant J.* 10 845–857. 10.1046/j.1365-313X.1996.10050845.x

[B84] Hieno A. Naznin H. A. Inaba-Hasegawa K. Yokogawa T. Hayami N. Nomoto M. (2019). Transcriptome analysis and identification of a transcriptional regulatory network in the response to H2O2. *Plant Physiol.* 180 1629–1646. 10.1104/pp.18.01426 31064811 PMC6752916

[B85] Hoekenga O. A. Maron L. G. Piñeros M. A. Cançado G. M. A. Shaff J. Kobayashi Y. (2006). AtALMT1, which encodes a malate transporter, is identified as one of several genes critical for aluminum tolerance in Arabidopsis. *Proc. Natl. Acad. Sci. U. S. A.* 103 9738–9743. 10.1073/pnas.0602868103 16740662 PMC1480476

[B86] Holbrook N. M. Shashidhar V. R. James R. A. Munns R. (2002). Stomatal control in tomato with ABA-deficient roots: response of grafted plants to soil drying. *J. Exp. Bot.* 53 1503–1514. 10.1093/jexbot/53.373.1503 12021298

[B87] Hou N. You J. Pang J. Xu M. Chen G. Yang Z. (2010). The accumulation and transport of abscisic acid insoybean (*Glycine max* L.) under aluminum stress. *Plant Soil* 330 127–137. 10.1007/s11104-009-0184-x

[B88] Hou Q. Ufer G. Bartels D. (2016). Lipid signalling in plant responses to abiotic stress. *Plant. Cell Environ.* 39 1029–1048. 10.1111/pce.12666 26510494

[B89] Hsieh L.-C. Lin S.-I. Shih A. C.-C. Chen J.-W. Lin W.-Y. Tseng C.-Y. (2009). Uncovering small RNA-mediated responses to phosphate deficiency in *Arabidopsis* by deep sequencing. *Plant Physiol.* 151 2120–2132. 10.1104/pp.109.147280 19854858 PMC2785986

[B90] Hu B. Wang W. Deng K. Li H. Zhang Z. Zhang L. (2015). MicroRNA399 is involved in multiple nutrient starvation responses in rice. *Front. Plant Sci.* 6:188. 10.3389/fpls.2015.00188 25852730 PMC4371656

[B91] Hu B. Zhu C. Li F. Tang J. Wang Y. Lin A. (2011). Leaf tip necrosis1 plays a pivotal role in the regulation of multiple phosphate starvation responses in rice. *Plant Physiol.* 156 1101–1115. 10.1104/pp.110.170209 21317339 PMC3135962

[B92] Hu H. Dai M. Yao J. Xiao B. Li X. Zhang Q. (2006). Overexpressing a NAM, ATAF, and CUC (NAC) transcription factor enhances drought resistance and salt tolerance in rice. *Proc. Natl. Acad. Sci. U.S.A.* 103 12987–12992. 10.1073/pnas.0604882103 16924117 PMC1559740

[B93] Huang C. F. Yamaji N. Chen Z. Ma J. F. (2012). A tonoplast-localized half-size ABC transporter is required for internal detoxification of aluminum in rice. *Plant J.* 69 857–867. 10.1111/j.1365-313X.2011.04837.x 22035218

[B94] Huang C. F. Yamaji N. Mitani N. Yano M. Nagamura Y. Ma J. F. (2009). A bacterial-type ABC transporter is involved in aluminum tolerance in rice. *Plant Cell* 21 655–667. 10.1105/tpc.108.064543 19244140 PMC2660611

[B95] Huang S. Gao J. You J. Liang Y. Guan K. Yan S. (2018). Identification of STOP1-like proteins associated with aluminum tolerance in sweet sorghum (*Sorghum bicolor* L.). *Front. Plant Sci.* 9:258. 10.3389/fpls.2018.00258 29541086 PMC5835670

[B96] Huang W.-J. Oo T. L. He H.-Y. Wang A.-Q. Zhan J. Li C.-Z. (2014). Aluminum induces rapidly mitochondria-dependent programmed cell death in Al-sensitive peanut root tips. *Bot. Stud.* 55:67. 10.1186/s40529-014-0067-1 PMC5432755 28510946

[B97] Hufnagel B. de Sousa S. M. Assis L. Guimaraes C. T. Leiser W. Azevedo G. C. (2014). Duplicate and conquer: Multiple homologs of PHOSPHORUS-STARVATION TOLERANCE1 enhance phosphorus acquisition and sorghum performance on low-phosphorus soils. *Plant Physiol.* 166 659–677. 10.1104/pp.114.243949 25189534 PMC4213096

[B98] Hufnagel B. Marques A. Soriano A. Marquès L. Divol F. Doumas P. (2020). High-quality genome sequence of white lupin provides insight into soil exploration and seed quality. *Nat. Commun.* 11:492. 10.1038/s41467-019-14197-9 31980615 PMC6981116

[B99] Ischebeck T. Seiler S. Heilmann I. (2010). At the poles across kingdoms: phosphoinositides and polar tip growth. *Protoplasma* 240 13–31. 10.1007/s00709-009-0093-0 20091065 PMC2841259

[B100] Ito H. Kobayashi Y. Yamamoto Y. Y. Koyama H. (2019). Characterization of NtSTOP1-regulating genes in tobacco under aluminum stress. *Soil Sci. Plant Nutr.* 65 251–258. 10.1080/00380768.2019.1603064

[B101] Iuchi S. Koyama H. Iuchi A. Kobayashi Y. Kitabayashi S. Kobayashi Y. (2007). Zinc finger protein STOP1 is critical for proton tolerance in *Arabidopsis* and coregulates a key gene in aluminum tolerance. *Proc. Natl. Acad. Sci. U.S.A.* 104 9900–9905. 10.1073/pnas.0700117104 17535918 PMC1887543

[B102] Jiang H.-X. Yang L.-T. Qi Y.-P. Lu Y.-B. Huang Z.-R. Chen L.-S. (2015). Root iTRAQ protein profile analysis of two *Citrus* species differing in aluminum-tolerance in response to long-term aluminum-toxicity. *BMC Genomics* 16:949. 10.1186/s12864-015-2133-9 26573913 PMC4647617

[B103] Jin J. F. Wang Z. Q. He Q. Y. Wang J. Y. Li P. F. Xu J. M. (2020). Genome-wide identification and expression analysis of the NAC transcription factor family in tomato (*Solanum lycopersicum*) during aluminum stress. *BMC Genomics* 21:288. 10.1186/s12864-020-6689-7 32264854 PMC7140551

[B104] Jones D. L. Kochian L. V. (1995). Aluminum inhibition of the inositol 1, 4, 5-trisphosphate signal transduction pathway in wheat roots: a role in aluminum toxicity? *Plant Cell* 7 1913–1922. 10.1105/tpc.7.11.1913 12242363 PMC161049

[B105] Jones H. G. Sharp C. S. Higgs K. H. (1987). Growth and water relations of wilty mutants of tomato (*Lycopersicon esculentum* Mill.). *J. Exp. Bot.* 38 1848–1856. 10.1093/jxb/38.11.1848 12432039

[B106] Jones R. J. Mansfield T. A. (1970). Suppression of stomatal opening in leaves treated with abscisic acid. *J. Exp. Bot.* 21 714–719. 10.1093/jxb/21.3.714 12432039

[B107] Joshi R. Wani S. H. Singh B. Bohra A. Dar Z. A. Lone A. A. (2016). Transcription factors and plants response to drought stress: current understanding and future directions. *Front. Plant Sci.* 7:1029. 10.3389/fpls.2016.01029 27471513 PMC4943945

[B108] Jung J. K. H. M. McCouch S. R. M. (2013). Getting to the roots of it: genetic and hormonal control of root architecture. *Front. Plant Sci.* 4:186. 10.3389/fpls.2013.00186 23785372 PMC3685011

[B109] Kang J. Hwang J.-U. Lee M. Kim Y.-Y. Assmann S. M. Martinoia E. (2010). PDR-type ABC transporter mediates cellular uptake of the phytohormone abscisic acid. *Proc. Natl. Acad. Sci. U.S.A.* 107 2355–2360. 10.1073/pnas.0909222107 20133880 PMC2836657

[B110] Kanneganti V. Gupta A. K. (2008). Wall associated kinases from plants—an overview. *Physiol. Mol. Biol. Plants* 14 109–118. 10.1007/s12298-008-0010-6 23572878 PMC3550657

[B111] Kanneganti V. Gupta A. K. (2011). RNAi mediated silencing of a wall associated kinase, OsiWAK1 in *Oryza sativa* results in impaired root development and sterility due to anther indehiscence. *Physiol. Mol. Biol. Plants* 17 65–77. 10.1007/s12298-011-0050-1 23572996 PMC3550565

[B112] Kasai M. Sasaki M. Tanakamaru S. Yamamoto Y. Matsumoto H. (1993). Possible involvement of abscisic acid in increases in activities of two vacuolar H+-pumps in barley roots under aluminum stress. *Plant Cell Physiol.* 34 1335–1338.

[B113] Kasai M. Sasaki M. Yamashita K. Yamamoto Y. Matsumoto H. (1995). “Increase of ATP-dependent H+ pump activity of tonoplast of barley roots by aluminium stress: possible involvement of abscisic acid for the regulation,” in *Plant-Soil Interactions at Low pH: Principles and Management*, eds Date R. A. Grundon N. J. Rayment G. E. Probert M. E. (Dordrecht: Springer), 341–344. 10.1007/978-94-011-0221-6_48

[B114] Kaur R. Singh K. Singh J. (2013). A root-specific wall-associated kinase gene, HvWAK1, regulates root growth and is highly divergent in barley and other cereals. *Funct. Integr. Genomics* 13 167–177. 10.1007/s10142-013-0310-y 23443578

[B115] Keerthisinghe G. Hocking P. J. Ryan P. R. Delhaize E. (1998). Effect of phosphorus supply on the formation and function of proteoid roots of white lupin (*Lupinus albus* L.). *Plant. Cell Environ.* 21 467–478. 10.1046/j.1365-3040.1998.00300.x

[B116] Kobayashi Y. Kobayashi Y. Sugimoto M. Lakshmanan V. Iuchi S. Kobayashi M. (2013a). Characterization of the complex regulation of AtALMT1 expression in response to phytohormones and other inducers. *Plant Physiol.* 162 732–740. 10.1104/pp.113.218065 23624855 PMC3668066

[B117] Kobayashi Y. Kobayashi Y. Watanabe T. Shaff J. E. Ohta H. Kochian L. V. (2013b). Molecular and physiological analysis of Al3+ and H+ rhizotoxicities at moderately acidic conditions. *Plant Physiol.* 163 180–192. 10.1104/pp.113.222893 23839867 PMC3762639

[B118] Kochian L. V. (1995). Cellular mechanisms of aluminum toxicity and resistance in plants. *Annu. Rev. Plant Biol.* 46 237–260. 10.1146/annurev.pp.46.060195.001321

[B119] Kochian L. V. Hoekenga O. A. Pineros M. A. (2004). How do crop plants tolerate acid soils? Mechanisms of aluminum tolerance and phosphorous efficiency. *Annu. Rev. Plant Biol.* 55 459–493. 10.1146/annurev.arplant.55.031903.141655 15377228

[B120] Kochian L. V. Piñeros M. A. Liu J. Magalhaes J. V. (2015). Plant adaptation to acid soils: the molecular basis for crop aluminum resistance. *Annu. Rev. Plant Biol.* 66 571–598. 10.1146/annurev-arplant-043014-114822 25621514

[B121] Kollmeier M. Felle H. H. Horst W. J. (2000). Genotypical differences in aluminum resistance of maize are expressed in the distal part of the transition zone. Is reduced basipetal auxin flow involved in inhibition of root elongation by aluminum? *Plant Physiol.* 122 945–956. 10.1104/pp.122.3.945 10712559 PMC58931

[B122] Kumar M. Chauhan A. S. Yusuf M. A. Sanyal I. Chauhan P. S. (2019). Transcriptome sequencing of chickpea (Cicer arietinum l.) genotypes for identification of drought-responsive genes under drought stress condition. *Plant Mol. Biol. Rep.* 37 186–203. 10.1007/s11105-019-01147-4

[B123] Kundu A. Das S. Basu S. Kobayashi Y. Kobayashi Y. Koyama H. (2019). GhSTOP1, a C2H2 type zinc finger transcription factor is essential for Aluminum and proton stress tolerance and lateral root initiation in cotton. *Plant Biol.* 21 35–44. 10.1111/plb.12895 30098101

[B124] Kuromori T. Miyaji T. Yabuuchi H. Shimizu H. Sugimoto E. Kamiya A. (2010). ABC transporter AtABCG25 is involved in abscisic acid transport and responses. *Proc. Natl. Acad. Sci. U.S.A.* 107 2361–2366. 10.1073/pnas.0912516107 20133881 PMC2836683

[B125] Kuromori T. Mizoi J. Umezawa T. Yamaguchi-Shinozaki K. Shinozaki K. (2014). Drought stress signaling network. *Mol. Biol.* 2 383–409. 10.1007/978-1-4614-7570-5_7

[B126] Kusunoki K. Nakano Y. Tanaka K. Sakata Y. Koyama H. Kobayashi Y. (2017). Transcriptomic variation among six *Arabidopsis thaliana* accessions identified several novel genes controlling aluminium tolerance. *Plant. Cell Environ.* 40 249–263. 10.1111/pce.12866 27861992

[B127] Larsen P. B. Geisler M. J. B. Jones C. A. Williams K. M. Cancel J. D. (2005). ALS3 encodes a phloem-localized ABC transporter-like protein that is required for aluminum tolerance in *Arabidopsis*. *Plant J.* 41 353–363. 10.1111/j.1365-313X.2004.02306.x 15659095

[B128] Lee D.-K. Chung P. J. Jeong J. S. Jang G. Bang S. W. Jung H. (2017). The rice OsNAC 6 transcription factor orchestrates multiple molecular mechanisms involving root structural adaptions and nicotianamine biosynthesis for drought tolerance. *Plant Biotechnol. J.* 15 754–764. 10.1111/pbi.12673 27892643 PMC5425393

[B129] Lee D.-K. Jung H. Jang G. Jeong J. S. Kim Y. S. Ha S.-H. (2016). Overexpression of the OsERF71 transcription factor alters rice root structure and drought resistance. *Plant Physiol.* 172 575–588. 10.1104/pp.16.00379 27382137 PMC5074616

[B130] Lee H. W. Kim N. Y. Lee D. J. Kim J. (2009). LBD18/ASL20 regulates lateral root formation in combination with LBD16/ASL18 downstream of ARF7 and ARF19 in *Arabidopsis*. *Plant Physiol.* 151 1377–1389. 10.1104/pp.109.143685 19717544 PMC2773067

[B131] Leng P. Zhao J. (2019). Transcription factors as molecular switches to regulate drought adaptation in maize. *Theor. Appl. Genet.* 133 1455–1465. 10.1007/s00122-019-03494-y 31807836

[B132] Li C. X. Yan J. Y. Ren J. Y. Sun L. Xu C. Li G. X. (2019). A WRKY transcription factor confers aluminum tolerance via regulation of cell wall modifying genes. *J. Integr. Plant Biol.* 62 1176–1192. 10.1111/jipb.12888 31729146

[B133] Li G. Z. Wang Z. Q. Yokosho K. Ding B. Fan W. Gong Q. Q. (2018). Transcription factor WRKY22 promotes aluminum tolerance via activation of OsFRDL4 expression and enhancement of citrate secretion in rice (*Oryza sativa*). *New Phytol.* 219 149–162. 10.1111/nph.15143 29658118

[B134] Li H. Han X. Liu X. Zhou M. Ren W. Zhao B. (2019). A leucine-rich repeat-receptor-like kinase gene SbER2–1 from sorghum (*Sorghum bicolor* L.) confers drought tolerance in maize. *BMC Genomics* 20:737. 10.1186/s12864-019-6143-x 31615416 PMC6794760

[B135] Li Z. Gao Q. Liu Y. He C. Zhang X. Zhang J. (2011). Overexpression of transcription factor ZmPTF1 improves low phosphate tolerance of maize by regulating carbon metabolism and root growth. *Planta* 233 1129–1143. 10.1007/s00425-011-1368-1 21312041

[B136] Li Z. Liu C. Zhang Y. Wang B. Ran Q. Zhang J. (2019). The bHLH family member ZmPTF1 regulates drought tolerance in maize by promoting root development and abscisic acid synthesis. *J. Exp. Bot.* 70 5471–5486. 10.1093/jxb/erz307 31267122 PMC6793450

[B137] Linzhou H. Yasir T. A. Phillips A. L. Hu Y.-G. (2013). Isolation and characterization of ERECTA genes and their expression patterns in common wheat (*Triticum aestivum* L.). *Aust. J. Crop Sci.* 7 381–390.

[B138] Liu G. Gao S. Tian H. Wu W. Robert H. S. Ding Z. (2016). Local transcriptional control of YUCCA regulates auxin promoted root-growth inhibition in response to aluminium stress in *Arabidopsis*. *PLoS Genet.* 12:e1006360. 10.1371/journal.pgen.1006360 27716807 PMC5065128

[B139] Liu J. Magalhaes J. V. Shaff J. Kochian L. V. (2009). Aluminum-activated citrate and malate transporters from the MATE and ALMT families function independently to confer *Arabidopsis aluminum* tolerance. *Plant J.* 57 389–399. 10.1111/j.1365-313X.2008.03696.x 18826429

[B140] López-Bucio J. Cruz-Ramírez A. Herrera-Estrella L. (2003). The role of nutrient availability in regulating root architecture. *Curr. Opin. Plant Biol.* 6 280–287. 10.1016/S1369-5266(03)00035-9 12753979

[B141] López-Bucio J. S. Salmerón-Barrera G. J. Ravelo-Ortega G. Raya-González J. León P. de la Cruz H. R. (2019). Mitogen-activated protein kinase 6 integrates phosphate and iron responses for indeterminate root growth in *Arabidopsis thaliana*. *Planta* 250 1177–1189. 10.1007/s00425-019-03212-4 31190117

[B142] Lou H. Q. Fan W. Jin J. F. Xu J. M. Chen W. W. Yang J. L. (2020). A NAC-type transcription factor confers aluminium resistance by regulating cell wall-associated receptor kinase 1 and cell wall pectin. *Plant Cell Environ.* 43 463–478. 10.1111/pce.13676 31713247

[B143] Loveys B. R. Kriedemann P. E. (1974). Internal control of stomatal physiology and photosynthesis. I. Stomatal regulation and associated changes in endogenous levels of abscisic and phaseic acids. *Funct. Plant Biol.* 1 407–415. 10.1071/pp9740407

[B144] Lv Q. Zhong Y. Wang Y. Wang Z. Zhang L. Shi J. (2014). SPX4 negatively regulates phosphate signaling and homeostasis through its interaction with PHR2 in rice. *Plant Cell* 26 1586–1597. 10.1105/tpc.114.123208 24692424 PMC4036573

[B145] Lynch J. P. (2011). Root phenes for enhanced soil exploration and phosphorus acquisition: tools for future crops. *Plant Physiol.* 156 1041–1049. 10.1104/pp.111.175414 21610180 PMC3135935

[B146] Ma J. F. Ryan P. R. Delhaize E. (2001). Aluminium tolerance in plants and the complexing role of organic acids. *Trends Plant Sci.* 6 273–278. 10.1016/s1360-1385(01)01961-6 11378470

[B147] Ma Y. Szostkiewicz I. Korte A. Moes D. Yang Y. Christmann A. (2009). Regulators of PP2C phosphatase activity function as abscisic acid sensors. *Science* 324 1064–1068. 19407143 10.1126/science.1172408

[B148] Magalhaes J. V. Liu J. Guimarães C. T. Lana U. G. P. Alves V. M. C. Wang Y. H. (2007). A gene in the multidrug and toxic compound extrusion (MATE) family confers aluminum tolerance in sorghum. *Nat. Genet.* 39 1156–1161. 10.1038/ng2074 17721535

[B149] Maierhofer T. Diekmann M. Offenborn J. N. Lind C. Bauer H. Hashimoto K. (2014). Site-and kinase-specific phosphorylation-mediated activation of SLAC1, a guard cell anion channel stimulated by abscisic acid. *Sci. Signal.* 7:ra86. 10.1126/scisignal.2005703 25205850

[B150] Marinho J. P. Kanamori N. Ferreira L. C. Fuganti-Pagliarini R. Carvalho J. de F. C. (2016). Characterization of molecular and physiological responses under water deficit of genetically modified soybean plants overexpressing the AtAREB1 transcription factor. *Plant Mol. Biol. Report.* 34 410–426. 10.1007/s11105-015-0928-0

[B151] Marschner H. (1995). Adaptation of plants to adverse chemical soil conditions. *Marschners Miner. Nutr. High. Plants* 2012 409–472. 10.1016/b978-0-12-384905-2.00017-0

[B152] Martín A. C. Del Pozo J. C. Iglesias J. Rubio V. Solano R. De La Peña A. (2000). Influence of cytokinins on the expression of phosphate starvation responsive genes in *Arabidopsis*. *Plant J.* 24 559–567. 10.1046/j.1365-313X.2000.00893.x 11123795

[B153] Masle J. Gilmore S. R. Farquhar G. D. (2005). The ERECTA gene regulates plant transpiration efficiency in *Arabidopsis*. *Nature* 436 866–870. 10.1038/nature03835 16007076

[B154] Meijer H. J. G. Munnik T. (2003). Phospholipid-based signaling in plants. *Annu. Rev. Plant Biol.* 54 265–306. 14502992 10.1146/annurev.arplant.54.031902.134748

[B155] Melo J. O. Martins L. G. C. Barros B. A. Pimenta M. R. Lana U. G. P. Duarte C. E. M. (2019). Repeat variants for the SbMATE transporter protect sorghum roots from aluminum toxicity by transcriptional interplay in cis and trans. *Proc. Natl. Acad. Sci. U.S.A.* 116 313–318. 10.1073/pnas.1808400115 30545913 PMC6320528

[B156] Mendes F. F. Guimarães L. J. M. Souza J. C. Guimarães P. E. O. Magalhaes J. V. Garcia A. A. F. (2014). Genetic architecture of phosphorus use efficiency in tropical maize cultivated in a low-P soil. *Crop Sci.* 54 1530–1538. 10.2135/cropsci2013.11.0755

[B157] Miller G. A. D. Suzuki N. Ciftci-Yilmaz S. Mittler R. O. N. (2010). Reactive oxygen species homeostasis and signalling during drought and salinity stresses. *Plant. Cell Environ.* 33 453–467. 10.1111/j.1365-3040.2009.02041.x 19712065

[B158] Mishra D. Shekhar S. Singh D. Chakraborty S. Chakraborty N. (2018). “Heat shock proteins and abiotic stress tolerance in plants,” in *Regulation of Heat Shock Protein Responses*, eds Asea A. Kaur P. (Cham: Springer), 41–69. 10.1007/978-3-319-74715-6_3

[B159] Mittler R. (2017). ROS are good. *Trends Plant Sci.* 22 11–19. 10.1016/j.tplants.2016.08.002 27666517

[B160] Miura K. Lee J. Gong Q. Ma S. Jin J. B. Yoo C. Y. (2011). SIZ1 Regulation of phosphate starvation-induced root architecture remodeling involves the control of auxin accumulation. *Plant Physiol.* 155 1000–1012. 10.1104/pp.110.165191 21156857 PMC3032448

[B161] Mora-Macías J. Ojeda-Rivera J. O. Gutiérrez-Alanís D. Yong-Villalobos L. Oropeza-Aburto A. Raya-González J. (2017). Malate-dependent Fe accumulation is a critical checkpoint in the root developmental response to low phosphate. *Proc. Natl. Acad. Sci. U.S.A.* 114 E3563–E3572. 10.1073/pnas.1701952114 28400510 PMC5410833

[B162] Müller J. Toev T. Heisters M. Teller J. Moore K. L. Hause G. (2015). Iron-dependent callose deposition adjusts root meristem maintenance to phosphate availability. *Dev. Cell* 33 216–230. 10.1016/j.devcel.2015.02.007 25898169

[B163] Munemasa S. Hauser F. Park J. Waadt R. Brandt B. Schroeder J. I. (2015). Mechanisms of abscisic acid-mediated control of stomatal aperture. *Curr. Opin. Plant Biol.* 28 154–162. 10.1016/j.pbi.2015.10.010 26599955 PMC4679528

[B164] Nakashima K. Takasaki H. Mizoi J. Shinozaki K. Yamaguchi-Shinozaki K. (2012). NAC transcription factors in plant abiotic stress responses. *Biochim. Biophys. Acta Gene Regul. Mech.* 1819 97–103. 10.1016/j.bbagrm.2011.10.005 22037288

[B165] Nakashima K. Yamaguchi-Shinozaki K. Shinozaki K. (2014). The transcriptional regulatory network in the drought response and its crosstalk in abiotic stress responses including drought, cold, and heat. *Front. Plant Sci.* 5:170. 10.3389/fpls.2014.00170 24904597 PMC4032904

[B166] Neumann G. Massonneau A. Langlade N. Dinkelaker B. Hengeler C. Römheld V. (2000). Physiological aspects of cluster root function and development in phosphorus-deficient white lupin (*Lupinus albus* L.). *Ann. Bot.* 85 909–919. 10.1006/anbo.2000.1135

[B167] Nishimura N. Hitomi K. Arvai A. S. Rambo R. P. Hitomi C. Cutler S. R. (2009). Structural mechanism of abscisic acid binding and signaling by dimeric PYR1. *Science* 326 1373–1379. 10.1126/science.1181829 19933100 PMC2835493

[B168] Oh J. E. Kwon Y. Kim J. H. Noh H. Hong S. W. Lee H. (2011). A dual role for MYB60 in stomatal regulation and root growth of *Arabidopsis thaliana* under drought stress. *Plant Mol. Biol.* 77 91–103. 10.1007/s11103-011-9796-7 21637967

[B169] Ohyama Y. Ito H. Kobayashi Y. Ikka T. Morita A. Kobayashi M. (2013). Characterization of AtSTOP1 orthologous genes in tobacco and other plant species. *Plant Physiol.* 162 1937–1946. 10.1104/pp.113.218958 23749850 PMC3729772

[B170] Ouyang X. Hong X. Zhao X. Zhang W. He X. Ma W. (2016). Knock out of the PHOSPHATE 2 gene TaPHO2-A1 improves phosphorus uptake and grain yield under low phosphorus conditions in common wheat. *Sci. Rep.* 6:29850. 10.1038/srep29850 27416927 PMC4945926

[B171] Overvoorde P. Fukaki H. Beeckman T. (2010). Auxin control of root development. *Cold Spring Harb. Perspect. Biol.* 2:a001537. 10.1101/cshperspect.a001537 PMC2869515 20516130

[B172] Park S.-Y. Fung P. Nishimura N. Jensen D. R. Fujii H. Zhao Y. (2009). Abscisic acid inhibits type 2C protein phosphatases via the PYR/PYL family of START proteins. *Science* 324 1068–1071. 19407142 10.1126/science.1173041 PMC2827199

[B173] Peñaloza E. Corcuera L. J. Martinez J. (2002). Spatial and temporal variation in citrate and malate exudation and tissue concentration as affected by P stress in roots of white lupin. *Plant Soil* 241 209–221. 10.1023/A:1016148222687

[B174] Peñaloza E. Muñoz G. Salvo-Garrido H. Silva H. Corcuera L. J. (2005). Phosphate deficiency regulates phosphoenolpyruvate carboxylase expression in proteoid root clusters of white lupin. *J. Exp. Bot.* 56 145–153. 10.1093/jxb/eri008 15501907

[B175] Petersson S. V. Johansson A. I. Kowalczyk M. Makoveychuk A. Wang J. Y. Moritz T. (2009). An auxin gradient and maximum in the Arabidopsis root apex shown by high-resolution cell-specific analysis of IAA distribution and synthesis. *Plant Cell* 21 1659–1668. 10.1105/tpc.109.066480 19491238 PMC2714926

[B176] Poirier Y. Thoma S. Somerville C. Schiefelbein J. (1991). A mutant of *Arabidopsis* deficient in xylem loading of phosphate. *Plant Physiol.* 97 1087–1093. 10.1104/pp.97.3.1087 16668493 PMC1081126

[B177] Poot-Poot W. Teresa Hernandez-Sotomayor S. M. (2011). Aluminum stress and its role in the phospholipid signaling pathway in plants and possible biotechnological applications. *IUBMB Life* 63 864–872. 10.1002/iub.550 21905199

[B178] Pougach K. Voet A. Kondrashov F. A. Voordeckers K. Christiaens J. F. Baying B. (2014). Duplication of a promiscuous transcription factor drives the emergence of a new regulatory network. *Nat. Commun.* 5 1–12. 10.1038/ncomms5868 PMC4172970 25204769

[B179] Puga M. I. Mateos I. Charukesi R. Wang Z. Franco-Zorrilla J. M. De Lorenzo L. (2014). SPX1 is a phosphate-dependent inhibitor of Phosphate Starvation response 1 in Arabidopsis. *Proc. Natl. Acad. Sci. U.S.A.* 111 14947–14952. 10.1073/pnas.1404654111 25271326 PMC4205628

[B180] Qiu Y. Yu D. (2009). Over-expression of the stress-induced OsWRKY45 enhances disease resistance and drought tolerance in *Arabidopsis*. *Environ. Exp. Bot.* 65 35–47. 10.1016/j.envexpbot.2008.07.002

[B181] Rabara R. C. Tripathi P. Rushton P. J. (2014). The potential of transcription factor-based genetic engineering in improving crop tolerance to drought. *Omi. A J. Integr. Biol.* 18 601–614. 10.1089/omi.2013.0177 25118806 PMC4175970

[B182] Ramírez M. Flores-Pacheco G. Reyes J. L. Álvarez A. L. Drevon J. J. Girard L. (2013). Two common bean genotypes with contrasting response to phosphorus deficiency show variations in the microRNA 399-mediated PvPHO2 regulation within the PvPHR1 signaling pathway. *Int. J. Mol. Sci.* 14 8328–8344. 10.3390/ijms14048328 23591845 PMC3645745

[B183] Ren X. Chen Z. Liu Y. Zhang H. Zhang M. Liu Q. (2010). ABO3, a WRKY transcription factor, mediates plant responses to abscisic acid and drought tolerance in *Arabidopsis*. *Plant J.* 63 417–429. 10.1111/j.1365-313X.2010.04248.x 20487379 PMC3117930

[B184] Reyna-Llorens I. Corrales I. Poschenrieder C. Barcelo J. Cruz-Ortega R. (2015). Both aluminum and ABA induce the expression of an ABC-like transporter gene (FeALS3) in the Al-tolerant species *Fagopyrum esculentum*. *Environ. Exp. Bot.* 111 74–82. 10.1016/j.envexpbot.2014.11.005

[B185] Rodríguez-Leal D. Lemmon Z. H. Man J. Bartlett M. E. Lippman Z. B. (2017). Engineering quantitative trait variation for crop improvement by genome editing. *Cell* 171 470–480. 10.1016/j.cell.2017.08.030 28919077

[B186] Rubio V. Linhares F. Solano R. Martín A. C. Iglesias J. Leyva A. (2001). A conserved MYB transcription factor involved in phosphate starvation signaling both in vascular plants and in unicellular algae. *Genes Dev.* 15 2122–2133. 10.1101/gad.204401 11511543 PMC312755

[B187] Rushton D. L. Tripathi P. Rabara R. C. Lin J. Ringler P. Boken A. K. (2012). WRKY transcription factors: Key components in abscisic acid signalling. *Plant Biotechnol. J.* 10 2–11. 10.1111/j.1467-7652.2011.00634.x 21696534

[B188] Ryan P. R. Delhaize E. Jones D. L. (2001). Function and mechanism of organic anion exudation from plant roots. *Annu. Rev. Plant Biol.* 52 527–560. 10.1146/annurev.arplant.52.1.527 11337408

[B189] Ryan P. R. Ditomaso J. M. Kochian L. V. (1993). Aluminium toxicity in roots: an investigation of spatial sensitivity and the role of the root cap. *J. Exp. Bot.* 44 437–446. 10.1093/jxb/44.2.437 12432039

[B190] Saab I. N. Sharp R. E. Pritchard J. Voetberg G. S. (1990). Increased endogenous abscisic acid maintains primary root growth and inhibits shoot growth of maize seedlings at low water potentials. *Plant Physiol.* 93 1329–1336. 10.1104/pp.93.4.1329 16667621 PMC1062676

[B191] Sadhukhan A. Enomoto T. Kobayashi Y. Watanabe T. Iuchi S. Kobayashi M. (2019). Sensitive to Proton Rhizotoxicity1 regulates salt and drought tolerance of Arabidopsis thaliana through transcriptional regulation of CIPK23. *Plant Cell Physiol.* 60 2113–2126. 10.1093/pcp/pcz120 31241160

[B192] Salvi S. Giuliani S. Ricciolini C. Carraro N. Maccaferri M. Presterl T. (2016). Two major quantitative trait loci controlling the number of seminal roots in maize co-map with the root developmental genes rtcs and rum1. *J. Exp. Bot.* 67 1149–1159. 10.1093/jxb/erw011 26880748 PMC4753855

[B193] Salvi S. Sponza G. Morgante M. Tomes D. Niu X. Fengler K. A. (2007). Conserved noncoding genomic sequences associated with a flowering-time quantitative trait locus in maize. *Proc. Natl. Acad. Sci. U.S.A.* 104 11376–11381. 10.1073/pnas.0704145104 17595297 PMC2040906

[B194] Sánchez-Calderón L. López-Bucio J. Chacón-López A. Cruz-Ramírez A. Nieto-Jacobo F. Dubrovsky J. G. (2005). Phosphate starvation induces a determinate developmental program in the roots of *Arabidopsis thaliana*. *Plant Cell Physiol.* 46 174–184. 10.1093/pcp/pci011 15659445

[B195] Santiago J. Dupeux F. Round A. Antoni R. Park S.-Y. Jamin M. (2009). The abscisic acid receptor PYR1 in complex with abscisic acid. *Nature* 462 665–668. 10.1038/nature08591 19898494

[B196] Sasaki T. Yamamoto Y. Ezaki B. Katsuhara M. Ahn S. J. Ryan P. R. (2004). A wheat gene encoding an aluminum-activated malate transporter. *Plant J.* 37 645–653. 10.1111/j.1365-313X.2003.01991.x 14871306

[B197] Sawaki Y. Iuchi S. Kobayashi Y. Kobayashi Y. Ikka T. Sakurai N. (2009). Stop1 regulates multiple genes that protect arabidopsis from proton and aluminum toxicities. *Plant Physiol.* 150 281–294. 10.1104/pp.108.134700 19321711 PMC2675709

[B198] Sawaki Y. Kobayashi Y. Kihara-Doi T. Nishikubo N. Kawazu T. Kobayashi M. (2014). Identification of a STOP1-like protein in Eucalyptus that regulates transcription of Al tolerance genes. *Plant Sci.* 223 8–15. 10.1016/j.plantsci.2014.02.011 24767110

[B199] Sbabou L. Bucciarelli B. Miller S. Liu J. Berhada F. Filali-Maltouf A. (2010). Molecular analysis of SCARECROW genes expressed in white lupin cluster roots. *J. Exp. Bot.* 61 1351–1363. 10.1093/jxb/erp400 20167612 PMC2837254

[B200] Scarpella E. Simons E. J. Meijer A. H. (2005). Multiple regulatory elements contribute to the vascular-specific expression of the rice HD-zip gene Oshox1 in *Arabidopsis*. *Plant Cell Physiol.* 46 1400–1410. 10.1093/pcp/pci153 15964905

[B201] Schmidt R. R. Weits D. A. Feulner C. F. J. van Dongen J. T. (2018). Oxygen sensing and integrative stress signaling in plants. *Plant Physiol.* 176 1131–1142. 10.1104/pp.17.01394 29162635 PMC5813526

[B202] Shahid M. Pourrut B. Dumat C. Nadeem M. Aslam M. Pinelli E. (2014). “Heavy-metal-induced reactive oxygen species: phytotoxicity and physicochemical changes in plants,” in *Reviews of Environmental Contamination and Toxicology*, Vol. 232 ed. Whitacre D. (Cham: Springer), 1–44. 10.1007/978-3-319-06746-9_1 24984833

[B203] Shang Y. Yan L. Liu Z. Q. Cao Z. Mei C. Xin Q. (2010). The Mg-chelatase H subunit of Arabidopsis antagonizes a group of WRKY transcription repressors to relieve ABA-responsive genes of inhibition. *Plant Cell* 22 1909–1935. 10.1105/tpc.110.073874 20543028 PMC2910980

[B204] Sharma P. Jha A. B. Dubey R. S. Pessarakli M. (2012). Reactive oxygen species, oxidative damage, and antioxidative defense mechanism in plants under stressful conditions. *J. Bot.* 2012:217037.

[B205] Sharp R. E. Davies W. J. (1989). “Regulation of growth and development of plants growing with a restricted supply of water,” in *Plants Under Stress: Biochemistry, Physiology and Ecology and Their Application to Plant Improvement Society for Experimental Biology Seminar Series*, eds Jones H. G. Flowers T. J. Jones M. B. E. (Cambridge: Cambridge University Press), 71–94. 10.1017/CBO9780511661587.006

[B206] Sharp R. E. Wu Y. Voetberg G. S. Saab I. N. LeNoble M. E. (1994). Confirmation that abscisic acid accumulation is required for maize primary root elongation at low water potentials. *J. Exp. Bot.* 45 1743–1751. 10.1093/jxb/45.special_issue.1743 12432039

[B207] Shen H. Hou N. Schlicht M. Wan Y. Mancuso S. Baluska F. (2008). Aluminium toxicity targets PIN2 in Arabidopsis root apices: effects on PIN2 endocytosis, vesicular recycling, and polar auxin transport. *Chin. Sci. Bull.* 53:2480 10.1007/s11434-008-0332-3

[B208] Shen H. Ligaba A. Yamaguchi M. Osawa H. Shibata K. Yan X. (2004). Effect of K-252a and abscisic acid on the efflux of citrate from soybean roots. *J. Exp. Bot.* 55 663–671. 10.1093/jxb/erh058 14754917

[B209] Shin H. Shin H.-S. Dewbre G. R. Harrison M. J. (2004). Phosphate transport in *Arabidopsis*: Pht1; 1 and Pht1; 4 play a major role in phosphate acquisition from both low-and high-phosphate environments. *Plant J.* 39 629–642. 15272879 10.1111/j.1365-313X.2004.02161.x

[B210] Shin R. Burch A. Y. Huppert K. A. Tiwari S. B. Murphy A. S. Guilfoyle T. J. (2007). The Arabidopsis transcription factor MYB77 modulates auxin signal transduction. *Plant Cell* 19 2440–2453. 10.1105/tpc.107.050963 17675404 PMC2002618

[B211] Singh D. Laxmi A. (2015). Transcriptional regulation of drought response: a tortuous network of transcriptional factors. *Front. Plant Sci.* 6:895. 10.3389/fpls.2015.00895 26579147 PMC4625044

[B212] Sivaguru M. Ezaki B. He Z. H. Tong H. Osawa H. Baluška F. (2003). Aluminum-induced gene expression and protein localization of a cell wall-associated receptor kinase in *Arabidopsis*. *Plant Physiol.* 132 2256–2266. 10.1104/pp.103.022129 12913180 PMC181309

[B213] Sivaguru M. Liu J. Kochian L. V. (2013). Targeted expression of Sb MATE in the root distal transition zone is responsible for sorghum aluminum resistance. *Plant J.* 76 297–307. 23865685 10.1111/tpj.12290

[B214] Skubacz A. Daszkowska-Golec A. Szarejko I. (2016). The role and regulation of ABI5 (ABA-Insensitive 5) in plant development, abiotic stress responses and phytohormone crosstalk. *Front. Plant Sci.* 7:1884. 10.3389/fpls.2016.01884 28018412 PMC5159420

[B215] Soyk S. Lemmon Z. H. Oved M. Fisher J. Liberatore K. L. Park S. J. (2017). Bypassing negative epistasis on yield in tomato imposed by a domestication gene. *Cell* 169 1142–1155. 10.1016/j.cell.2017.04.032 28528644

[B216] Spollen W. G. LeNoble M. E. Samuels T. D. Bernstein N. Sharp R. E. (2000). Abscisic acid accumulation maintains maize primary root elongation at low water potentials by restricting ethylene production. *Plant Physiol.* 122 967–976. 10.1104/pp.122.3.967 10712561 PMC58933

[B217] Su T. Xu Q. Zhang F. C. Chen Y. Li L. Q. Wu W. H. (2015). WRKY42 modulates phosphate homeostasis through regulating phosphate translocation and acquisition in *Arabidopsis*. *Plant Physiol.* 167 1579–1591. 10.1104/pp.114.253799 25733771 PMC4378159

[B218] Sun P. Tian Q.-Y. Chen J. Zhang W.-H. (2010). Aluminium-induced inhibition of root elongation in Arabidopsis is mediated by ethylene and auxin. *J. Exp. Bot.* 61 347–356. 10.1093/jxb/erp306 19858117 PMC2803203

[B219] Sun X. Wang Y. Sui N. (2018). Transcriptional regulation of bHLH during plant response to stress. *Biochem. Biophys. Res. Commun.* 503 397–401. 10.1016/j.bbrc.2018.07.123 30057319

[B220] Suzuki N. Rizhsky L. Liang H. Shuman J. Shulaev V. Mittler R. (2005). Enhanced tolerance to environmental stress in transgenic plants expressing the transcriptional coactivator multiprotein bridging factor 1c. *Plant Physiol.* 139 1313–1322. 10.1104/pp.105.070110 16244138 PMC1283768

[B221] Takahashi F. Suzuki T. Osakabe Y. Betsuyaku S. Kondo Y. Dohmae N. (2018). A small peptide modulates stomatal control via abscisic acid in long-distance signalling. *Nature* 556 235–238. 10.1038/s41586-018-0009-2 29618812

[B222] Taramino G. Sauer M. Stauffer J. L. Multani D. Niu X. Sakai H. (2007). The maize (Zea mays L.) RTCS gene encodes a LOB domain protein that is a key regulator of embryonic seminal and post-embryonic shoot-borne root initiation. *Plant J.* 50 649–659. 10.1111/j.1365-313X.2007.03075.x 17425722

[B223] Thole J. M. Nielsen E. (2008). Phosphoinositides in plants: novel functions in membrane trafficking. *Curr. Opin. Plant Biol.* 11 620–631. 10.1016/j.pbi.2008.10.010 19028349

[B224] Thompson A. J. Mulholland B. J. Jackson A. C. McKee J. M. T. Hilton H. W. Symonds R. C. (2007). Regulation and manipulation of ABA biosynthesis in roots. *Plant. Cell Environ.* 30 67–78. 10.1111/j.1365-3040.2006.01606.x 17177877

[B225] Tokizawa M. Kobayashi Y. Saito T. Kobayashi M. Iuchi S. Nomoto M. (2015). Sensitive to Proton Rhizotoxicity1, calmodulin binding transcription activator2, and other transcription factors are involved in Aluminum-Activated Malate Transporter1 expression. *Plant Physiol.* 167 991–1003. 10.1104/pp.114.256552 25627216 PMC4348791

[B226] Tran L. S. P. Nakashima K. Sakuma Y. Simpson S. D. Fujita Y. Maruyama K. (2004). Isolation and functional analysis of arabidopsis stress-inducible NAC transcription factors that bind to a drought-responsive cis-element in the early responsive to dehydration stress 1 promoter. *Plant Cell* 16 2481–2498. 10.1105/tpc.104.022699 15319476 PMC520947

[B227] Tsutsui T. Yamaji N. Ma J. F. (2011). Identification of a cis-acting element of ART1, a C2H2-type zinc-finger transcription factor for aluminum tolerance in rice. *Plant Physiol.* 156 925–931. 10.1104/pp.111.175802 21502187 PMC3177286

[B228] Upadhyay N. Kar D. Datta S. (2020). A multidrug and toxic compound extrusion (MATE) transporter modulates auxin levels in root to regulate root development and promotes aluminium tolerance. *Plant. Cell Environ.* 43 745–759. 10.1111/pce.13658 31677167

[B229] Van Breusegem F. Dat J. F. (2006). Reactive oxygen species in plant cell death. *Plant Physiol.* 141 384–390. 16760492 10.1104/pp.106.078295 PMC1475453

[B230] Von Uexküll H. R. Mutert E. (1995). Global extent, development and economic impact of acid soils. *Plant Soil* 171 1–15. 10.1007/bf00009558

[B231] Wang H. Sun R. Cao Y. Pei W. Sun Y. Zhou H. (2015). OsSIZ1, a SUMO E3 ligase gene, is involved in the regulation of the responses to phosphate and nitrogen in rice. *Plant Cell Physiol.* 56 2381–2395. 10.1093/pcp/pcv162 26615033

[B232] Wang H. Xu Q. Kong Y. H. Chen Y. Duan J. Y. Wu W. H. (2014). Arabidopsis WRKY45 transcription factor activates Phosphate transporter1;1 expression in response to phosphate starvation. *Plant Physiol.* 164 2020–2029. 10.1104/pp.113.235077 24586044 PMC3982759

[B233] Wang X. Wang Z. Zheng Z. Dong J. Song L. Sui L. (2019). Genetic dissection of Fe-Dependent signaling in root developmental responses to phosphate deficiency. *Plant Physiol.* 179 300–316. 10.1104/pp.18.00907 30420567 PMC6324241

[B234] Wang Y.-S. Yang Z.-M. (2005). Nitric oxide reduces aluminum toxicity by preventing oxidative stress in the roots of *Cassia tora* L. *Plant Cell Physiol.* 46 1915–1923. 10.1093/pcp/pci202 16179356

[B235] Waszczak C. Carmody M. Kangasjärvi J. (2018). Reactive oxygen species in plant signaling. *Annu. Rev. Plant Biol.* 69 209–236. 29489394 10.1146/annurev-arplant-042817-040322

[B236] Wilkinson S. (1999). pH as a stress signal. *Plant Growth Regul.* 29 87–99.

[B237] Wilkinson S. Davies W. J. (1997). Xylem sap pH increase: a drought signal received at the apoplastic face of the guard cell that involves the suppression of saturable abscisic acid uptake by the epidermal symplast. *Plant Physiol.* 113 559–573. 10.1104/pp.113.2.559 12223626 PMC158172

[B238] Wiśniewska J. Xu J. Seifertová D. Brewer P. B. Ružička K. Blilou I. (2006). Polar PIN localization directs auxin flow in plants. *Science* 312:883. 10.1126/science.1121356 16601151

[B239] Wissuwa M. Wegner J. Ae N. Yano M. (2002). Substitution mapping of Pup1: a major QTL increasing phosphorus uptake of rice from a phosphorus-deficient soil. *Theor. Appl. Genet.* 105 890–897. 10.1007/s00122-002-1051-9 12582914

[B240] Wright S. T. C. (1980). The effect of plant growth regulator treatments on the levels of ethylene emanating from excised turgid and wilted wheat leaves. *Planta* 148 381–388. 10.1007/bf00388127 24310142

[B241] Wu D. Shen H. Yokawa K. Baluška F. (2014). Alleviation of aluminium-induced cell rigidity by overexpression of OsPIN2 in rice roots. *J. Exp. Bot.* 65 5305–5315. 10.1093/jxb/eru292 25053643 PMC4157713

[B242] Wu D. Shen H. Yokawa K. Baluška F. (2015). Overexpressing OsPIN2 enhances aluminium internalization by elevating vesicular trafficking in rice root apex. *J. Exp. Bot.* 66 6791–6801. 10.1093/jxb/erv385 26254327 PMC4623688

[B243] Wu L. Sadhukhan A. Kobayashi Y. Ogo N. Tokizawa M. Agrahari R. K. (2019). Involvement of phosphatidylinositol metabolism in aluminum-induced malate secretion in *Arabidopsis*. *J. Exp. Bot.* 70 3329–3342. 10.1093/jxb/erz179 30977815

[B244] Wu W. Lin Y. Chen Q. Peng W. Peng J. Tian J. (2018). Functional conservation and divergence of soybean GmSTOP1 members in proton and aluminum tolerance. *Front. Plant Sci.* 9:570. 10.3389/fpls.2018.00570 29755502 PMC5932199

[B245] Xia J. Yamaji N. Kasai T. Ma J. F. (2010). Plasma membrane-localized transporter for aluminum in rice. *Proc. Natl. Acad. Sci. U.S.A.* 107 18381–18385. 10.1073/pnas.1004949107 20937890 PMC2972927

[B246] Xia J. Yamaji N. Ma J. F. (2013). A plasma membrane-localized small peptide is involved in rice aluminum tolerance. *Plant J.* 76 345–355. 10.1111/tpj.12296 23888867

[B247] Xu C. Tai H. Saleem M. Ludwig Y. Majer C. Berendzen K. W. (2015). Cooperative action of the paralogous maize lateral organ boundaries (LOB) domain proteins RTCS and RTCL in shoot-borne root formation. *New Phytol.* 207 1123–1133. 10.1111/nph.13420 25902765

[B248] Yamaji N. Huang C. F. Nagao S. Yano M. Sato Y. Nagamura Y. (2009). A zinc finger transcription factor ART1 regulates multiple genes implicated in aluminum tolerance in rice. *Plant Cell* 21 3339–3349. 10.1105/tpc.109.070771 19880795 PMC2782276

[B249] Yamamoto Y. Kobayashi Y. Devi S. R. Rikiishi S. Matsumoto H. (2002). Aluminum toxicity is associated with mitochondrial dysfunction and the production of reactive oxygen species in plant cells. *Plant Physiol.* 128 63–72. 10.1104/pp.010417 11788753 PMC148944

[B250] Yang J. L. Li Y. Y. Zhang Y. J. Zhang S. S. Wu Y. R. Wu P. (2008). Cell wall polysaccharides are specifically involved in the exclusion of aluminum from the rice root apex. *Plant Physiol.* 146 602–611. 10.1104/pp.107.111989 18083797 PMC2245838

[B251] Yang L.-T. Chen L.-S. Peng H.-Y. Guo P. Wang P. Ma C.-L. (2012a). Organic acid metabolism in *Citrus grandis* leaves and roots is differently affected by nitric oxide and aluminum interactions. *Sci. Hortic.* 133 40–46. 10.1016/j.scienta.2011.10.011

[B252] Yang L.-T. Qi Y.-P. Chen L.-S. Sang W. Lin X.-J. Wu Y.-L. (2012b). Nitric oxide protects sour pummelo (*Citrus grandis*) seedlings against aluminum-induced inhibition of growth and photosynthesis. *Environ. Exp. Bot.* 82 1–13. 10.1016/j.envexpbot.2012.03.004

[B253] Yang Z. Chi X. Guo F. Jin X. Luo H. Hawar A. (2020a). SbWRKY30 enhances the drought tolerance of plants and regulates a drought stress-responsive gene, SbRD19, in sorghum. *J. Plant Physiol.* 246–247 153142. 10.1016/j.jplph.2020.153142 32112957

[B254] Yang Z. Yang J. Wang Y. Wang F. Mao W. He Q. (2020b). PROTEIN PHOSPHATASE95 regulates phosphate homeostasis by affecting phosphate transporter trafficking in rice. *Plant Cell* 32 740–757. 10.1105/tpc.19.00685 31919298 PMC7054036

[B255] Yang Z.-B. Geng X. He C. Zhang F. Wang R. Horst W. J. (2014). TAA1-regulated local auxin biosynthesis in the root-apex transition zone mediates the aluminum-induced inhibition of root growth in *Arabidopsis*. *Plant Cell* 26 2889–2904. 10.1105/tpc.114.127993 25052716 PMC4145121

[B256] Yao Y. He R. J. Xie Q. L. Zhao X. H. Deng X. M. He J. B. (2017). ETHYLENE RESPONSE FACTOR 74 (ERF74) plays an essential role in controlling a respiratory burst oxidase homolog D (RbohD)-dependent mechanism in response to different stresses in *Arabidopsis*. *New Phytol.* 213 1667–1681. 10.1111/nph.14278 28164334

[B257] Yi K. Wu Z. Zhou J. Du L. Guo L. Wu Y. (2005). OsPTF1, a novel transcription factor involved in tolerance to phosphate starvation in rice. *Plant Physiol.* 138 2087–2096. 10.1104/pp.105.063115 16006597 PMC1183397

[B258] Yokosho K. Yamaji N. Fujii-Kashino M. Ma J. F. (2016). Retrotransposon-mediated aluminum tolerance through enhanced expression of the citrate transporter OsFRDL4. *Plant Physiol.* 172 2327–2336. 10.1104/pp.16.01214 27744299 PMC5129714

[B259] Yokosho K. Yamaji N. Ma J. F. (2011). An Al-inducible MATE gene is involved in external detoxification of Al in rice. *Plant J.* 68 1061–1069. 10.1111/j.1365-313X.2011.04757.x 21880027

[B260] Zhang J. Davies W. J. (1989). Abscisic acid produced in dehydrating roots may enable the plant to measure the water status of the soil. *Plant. Cell Environ.* 12 73–81. 10.1111/j.1365-3040.1989.tb01918.x

[B261] Zhang J. Davies W. J. (1990a). Changes in the concentration of ABA in xylem sap as a function of changing soil water status can account for changes in leaf conductance and growth. *Plant. Cell Environ.* 13 277–285. 10.1111/j.1365-3040.1990.tb01312.x

[B262] Zhang J. Davies W. J. (1990b). Does ABA in the xylem control the rate of leaf growth in soil-dried maize and sunflower plants? *J. Exp. Bot.* 41 1125–1132. 10.1093/jxb/41.9.1125 12432039

[B263] Zhang J. Schurr U. Davies W. J. (1987). Control of stomatal behaviour by abscisic acid which apparently originates in the roots. *J. Exp. Bot.* 38 1174–1181. 10.1093/jxb/38.7.1174 12432039

[B264] Zhang Y. Zhang J. Guo J. Zhou F. Singh S. Xu X. (2019). F-box protein RAE1 regulates the stability of the aluminum-resistance transcription factor STOP1 in Arabidopsis. *Proc. Natl. Acad. Sci. U.S.A.* 116 319–327. 10.1073/pnas.1814426116 30559192 PMC6320511

[B265] Zhang Z. Wang H. Wang X. Bi Y. (2011). Nitric oxide enhances aluminum tolerance by affecting cell wall polysaccharides in rice roots. *Plant Cell Rep.* 30:1701. 10.1007/s00299-011-1078-y 21553108

[B266] Zhang Z. Zheng Y. Ham B.-K. Chen J. Yoshida A. Kochian L. V. (2016). Vascular-mediated signalling involved in early phosphate stress response in plants. *Nat. Plants* 2 1–9. 10.1081/e-epcs-120010640 27249565

[B267] Zhang Z. Zheng Y. Ham B.-K. Zhang S. Fei Z. Lucas W. J. (2019). Plant lncRNAs are enriched in and move systemically through the phloem in response to phosphate deficiency. *J. Integr. Plant Biol.* 61 492–508. 10.1111/jipb.12715 30171742

[B268] Zhao Y. Xing L. Wang X. Hou Y.-J. Gao J. Wang P. (2014). The ABA receptor PYL8 promotes lateral root growth by enhancing MYB77-dependent transcription of auxin-responsive genes. *Sci. Signal.* 7 ra53—ra53. 10.1126/scisignal.2005051 PMC4298826 24894996

[B269] Zhen Y. Qi J.-L. Wang S.-S. Su J. Xu G.-H. Zhang M.-S. (2007). Comparative proteome analysis of differentially expressed proteins induced by Al toxicity in soybean. *Physiol. Plant* 131 542–554. 10.1111/j.1399-3054.2007.00979.x 18251846

[B270] Zheng J. Yang Z. Madgwick P. J. Carmo-Silva E. Parry M. A. J. Hu Y.-G. (2015). TaER expression is associated with transpiration efficiency traits and yield in bread wheat. *PLoS One* 10:e0128415. 10.1371/journal.pone.0128415 26047019 PMC4457575

[B271] Zhu J. Brown K. M. Lynch J. P. (2010). Root cortical aerenchyma improves the drought tolerance of maize (Zea mays L.). *Plant Cell Environ.* 33 740–749. 20519019 10.1111/j.1365-3040.2009.02099.x

[B272] Zhu X. F. Wan J. X. Sun Y. Shi Y. Z. Braam J. Li G. X. (2014). Xyloglucan endotransglucosylase-hydrolase17 interacts with xyloglucan endotransglucosylase-hydrolase31 to confer xyloglucan endotransglucosylase action and affect aluminum sensitivity in arabidopsis. *Plant Physiol.* 165 1566–1574. 10.1104/pp.114.243790 24948835 PMC4119039

